# Study of the Integrated Immune Response Induced by an Inactivated EV71 Vaccine

**DOI:** 10.1371/journal.pone.0054451

**Published:** 2013-01-23

**Authors:** Longding Liu, Ying Zhang, Jingjing Wang, Hongling Zhao, Li Jiang, Yanchun Che, Haijin Shi, Rongcheng Li, Zhaojun Mo, Teng Huang, Zhenglun Liang, Qunying Mao, Lichun Wang, Chenghong Dong, Yun Liao, Lei Guo, Erxia Yang, Jing Pu, Lei Yue, Zhenxin Zhou, Qihan Li

**Affiliations:** 1 Institute of Medical Biology, Chinese Academy of Medicine Science, Peking Union Medical College, Kunming, People’s Republic of China; 2 Guangxi Province Centers for Disease Control and Prevention, Nanning, People’s Republic of China; 3 National Institutes for Food and Drug Control, Beijing, People’s Republic of China; University of Alabama at Birmingham, United States of America

## Abstract

Enterovirus 71 (EV71), a major causative agent of hand-foot-and-mouth disease (HFMD), causes outbreaks among children in the Asia-Pacific region. A vaccine is urgently needed. Based on successful pre-clinical work, phase I and II clinical trials of an inactivated EV71 vaccine, which included the participants of 288 and 660 respectively, have been conducted. In the present study, the immune response and the correlated modulation of gene expression in the peripheral blood mononuclear cells (PBMCs) of 30 infants (6 to 11 months) immunized with this vaccine or placebo and consented to join this study in the phase II clinical trial were analyzed. The results showed significantly greater neutralizing antibody and specific T cell responses in vaccine group after two inoculations on days 0 and 28. Additionally, more than 600 functional genes that were up- or down-regulated in PBMCs were identified by the microarray assay, and these genes included 68 genes associated with the immune response in vaccine group. These results emphasize the gene expression profile of the immune system in response to an inactivated EV71 vaccine in humans and confirmed that such an immune response was generated as the result of the positive mobilization of the immune system. Furthermore, the immune response was not accompanied by the development of a remarkable inflammatory response.

*Clinical Trial Registration: NCT01391494 and NCT01512706.*

## Introduction

Hand-foot-and-mouth disease (HFMD) is an acute infectious disease that poses a high risk to children’s health. As demonstrated in recent studies, enterovirus 71 (EV71) is a common etiological agent of HFMD [1], especially among fatal cases [2]. Based on the published epidemiological and clinical etiological data from recent years, approximately 80% to 85% of the pathogens isolated from patients who died from HFMD were identified as EV71 [3], [4]; in another study, that percentage was more than 95% for fatal cases [5], [6]. Therefore, an EV71 vaccine is necessary to decrease the number of fatal HFMD cases [2], [7]. Several candidate inactivated EV71 vaccines have undergone clinical trials [8]–[12]. However, the immunopathogenesis of EV71 infection in fatal cases of HFMD is not completely clear, and etiological characterization of the viral infection in conjunction with an analysis of the immunological effects of the virus is necessary. The evaluation of the safety and efficacy of the candidate EV71 vaccines used in clinical trials should be thorough.

The immunological analysis of the response to viral vaccines using the neutralizing antibody response as the major indicator is based on experiential consensus with respect to certain classical viral vaccines [13]–[15]. For example, this indicator has been used successfully to evaluate the efficacy of the poliovirus and hepatitis A virus vaccines worldwide [16], [17]. However, in the systematic biological studies of vaccines in which gene and protein expression profiling were used as the major approach to understand the systemic immune response, antibody response has been demonstrated to correlate well with the modulation of some genes associated with the function of the immune system [18]–[20]. These genes include some genes that are responsible for the inflammatory response and other genes that are responsible for regulating the variable integrated function of the immune system [20]. Thus, when evaluating the protective effects of the candidate EV71 vaccine using the classical test for specific virus-neutralizing antibodies, the inflammatory response after vaccination and its immunopathological significance should be strongly emphasized because the pathogenesis of EV71 infection is not completely clear. In our previous report on the EV71 vaccination of rhesus monkeys, the dose of the vaccine was shown to affect the balance between the Th1 and Th2 immune responses, which indicates that the Th1/Th2 response induced by different doses of EV71 inactivated vaccine tends to vary upon viral challenge [21]. In addition, the levels of some proinflammatory cytokines, such as interferon gamma (IFN-γ) and tumor necrosis factor alpha (TNF-α), are higher in fatal cases of EV71 infection [22]. These data underscore the importance of analyzing the immune response induced by the EV71 inactivated vaccine when evaluating the safety of the vaccine in a clinical trial. In the current study, we systemically analyzed and correlated the immune response and the modulation of genes in peripheral blood mononuclear cells (PBMCs) from volunteers who were immunized with the inactivated EV71 vaccine. These vaccine recipients belonged to specifically designated experimental cohorts in a phase II clinical trial. The results showed a significant increase in antibody levels and enhanced specific T cell responses in the peripheral blood specimens collected after primary and booster immunizations of infant subjects with an average age of 6 to 11 months. In addition, functional associations of the genes in PBMCs that were up- and down-regulated after vaccination were identified. These results appear to be the first to emphasize the gene expression profile of the immune system in response to the immunization of humans with an inactivated EV71 vaccine. The results of this study may eventually provide evidence for the safety of this EV71 vaccine and its ability to protect against the abnormal immunopathogenesis of EV71 infection.

## Methods

### Preparation and Quality Control of the Inactivated EV71 Vaccine Prepared from Human Diploid Cells

The inactivated EV71 vaccine was prepared in human diploid cells according to a published protocol [23]. Briefly, the viral strain of EV71 C4 sub-genotype was isolated from a male child with severe HFMD in Fuyang, China, in 2008 [24]. Based upon the analysis of the biological characteristics and genetic properties, the virus is adapted to grow in human diploid cells (KMB-17 strain). Subsequent immunological and safety analyses were performed [25]. In parallel, the vaccine samples for use in the clinical trials were prepared in compliance with relevant quality criteria. Its specification was 320 EU/dose (100 U/dose) with Al(OH)_3_ and was certificated by the National Institutes for Food and Drug Control (NICFDC) with lot number SHBM201001138 [26]. The placebo, with a lot number of SZBM201100583, was antigen of 0 U/dose with Al(OH)_3_. The immunization regimen was primary immunization followed by a booster 4 weeks later.

### Vaccination of Human Subjects and Subsequent Collection of Blood Samples

According to the principles of National Good Clinical Practice (GCP) and the Declaration of Helsinki, the inactivated EV71 vaccine was approved by the State Food and Drug Administration (SFDA) for clinical trials (NO.2010L05009) [27]. The clinical protocols were registered at ClinicalTrials.gov under the numbers NCT01391494 and NCT01512706 for the phase I and II trials, respectively [8], [9]. The trials were reviewed and approved by the Human Subjects Review Committee of the Institute of Medical Biology and the Ethics Committee of the Guangxi Clinical Trial Base for each study site under the document number IRB00001594.

The vaccine inoculation group and the placebo control group, originated from the cohort of phase II clinical trial, consisted of 20 subjects (aged from 6–11 months, average: 9.6 months) and 10 subjects (aged 6–11 months, average: 9.1 months) respectively. The subjects were selected for this experiment because their parents or guardians agreed to join this experiment. The basic characterizations of all of the participants are described in Table S1. The trial was randomized, double-blinded and placebo-controlled, and all of the parents or guardians of the subjects were required to sign an informed consent explaining the experiment. The vaccine was administered intramuscularly on day 0 and boosted on day 28. Between 2.0 and 2.5 ml of blood was taken from a vein before the primary immunization and 28 days after the booster. Of this blood, 0.5 ml was used to prepare serum for antibody detection, and the remaining 1.5–2.0 ml was treated with an anticoagulant prior to the extraction of total RNA from the PBMCs using a whole blood RNA extraction kit (Qiagen, GmBH, Germany) according to the manufacturer’s instructions. The RNA Integrity Number (RIN) was also evaluated to inspect RNA integrity using an Agilent Bioanalyzer 2100 (Agilent, CA, USA). The extracted RNA was temporarily frozen in 95% ethanol until further testing. 0.5 ml of blood was collected at 11 months after the booster immunization and used to prepare serum for the detection of neutralizing antibody.

### Microarray Screening of RNA Samples

The Whole Human Genome Microarray (Agilent, CA, USA) was chosen to screen for inactivated EV71 vaccine-modulated genes in human PBMC. GeneChip microarray experiments were conducted at the National Engineering Center for Biochip in Shanghai, China, according to the procedures in the Agilent technical manual. After hybridization, the microarrays were scanned in the Agilent Microarray Scanner (Cat# G2565CA, Agilent, CA, USA), and the raw data were obtained by Feature Extraction Software 10.7 (Agilent, CA, USA) and normalized using the Quantile algorithm in Gene Spring 11.0 (Agilent, CA, USA). The Normalization Value was set to 1. Only when the signal ratio value was greater than 2 was the corresponding gene regarded as up-regulated. Genes with ratios of less than 0.5 were regarded as down-regulated. To control the false discovery rate, the q value of all genes used in this analysis was assessed <0.05.

### Viral Neutralizing Antibody Assay

EV71 neutralizing antibody testing was performed according to a standard protocol [25], [28]. Briefly, diluted serum was mixed with equal volume of medium containing 500–1000 CCID_50_ of EV71 in 96-well plates, incubated and then added to confluent Vero cells. The cellular pathogenic effect (CPE) caused by viral infection was examined. The highest dilution was considered to be the neutralization titer.

### IFN-γ specific Elispot Assay

A standard Elispot assay was performed as described in previously published reports [25]. Briefly, a 96-well polyvinylidene difluoride-backed plate was pre-coated with anti-IFN-γ mAb, incubated overnight, and blocked for 1 h at 37°C. Wells containing PBMCs of predetermined density and stimulating peptides (10 µg/ml) were incubated at 37°C for another 24 h. Next, the cells were removed, and the colors were developed according to the manufacturer’s protocol. The colored spots were counted with an automated Elispot reader (CTL, OH, USA). The spot-forming cells (SFCs) represent EV71 epitope-specific IFN-γ-producing T cells.

### Flow Cytometry-based Cytometric Bead Array Analysis

Serum from the subjects was collected to be used in detection assays targeting IL-1b, IL-2, IL-4, IL-5, IL-6, IL-8, IL-10, IL12p70, TNF-α and IFN-γ. These assays were conducted simultaneously using a human Th1/Th2 cytokine cytometric bead array kit (BD Biosciences, San Diego, CA, USA) [29]. The assay was conducted following the manufacturer’s protocol.

### Real-time RT-PCR

The gene expression levels were measured by real-time RT-PCR, as previously described [30]. The primers are listed in Table S2. Briefly, total RNA was extracted from the PBMCs as described above. For quantification, a single-tube RT-PCR assay was performed using the TaqMan 1-step RT-PCR Master Mix in a 7500 Fast Real-time RT-PCR system (Applied Biosystems, Foster City, CA, USA). The following protocol was used for all PCR assays: 5 min at 42°C and 10 s at 95°C, followed by 40 cycles at 95°C for 5 s and 60°C for 30 s.

### Statistical Analysis

The predicted outcome of this experiment was based on all of the participants who received two doses of the vaccine and demonstrated an elevated immune response against the EV71 antigen, which was demonstrated through an increase in neutralizing antibody titers associated with the modulation of systematic gene expression. The neutralizing antibody levels and fold changes in gene expression were used to evaluate the immune response induced by the vaccine under the assumption that there were significant differences for both of these indicators between the vaccine group and the placebo group. The response of the vaccinated subjects with a 95% CI were calculated by comparing the two groups. Changes in the cytokine levels in the peripheral blood and the genes related to inflammation in the two groups were compared to detect any differences. The statistical analysis was performed using SPSS 13.0 software (SPSS Inc, IL, USA). The difference between the two groups was evaluated using one-way ANOVA. A P-value of <0.05 was considered to be significant.

## Results

### The Inactivated EV71 Vaccine Induced a Specific Immune Response Against an EV71 Antigen

**Figure 1:**
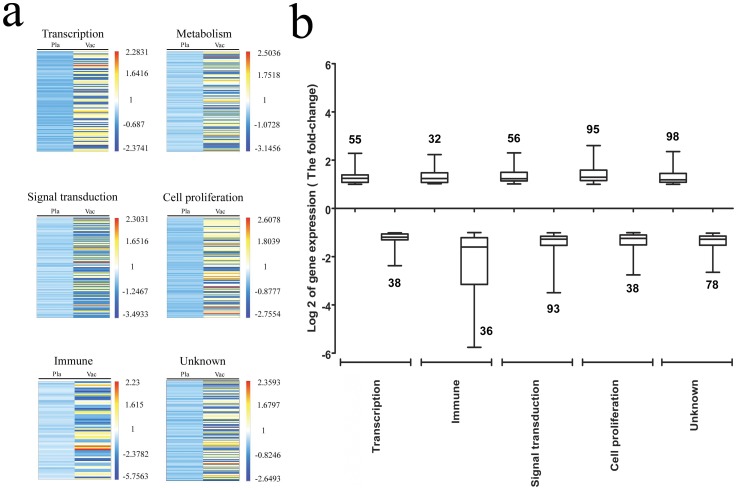
Vaccination with the inactivated EV71 vaccine modulated gene expression. A global view of gene modulation in the PBMCs of vaccinated infants (Vac, n = 20), 28 days after the vaccine booster, compared to the placebo cohort (Pla, n = 10) was determined by microarray analysis. The genes were classified according to their function. A heat map representation (a) and principal component analysis (PCA) (b) generated using the significantly modulated genes from at least one comparison versus day 0 are shown. (a) The color scale shows the significantly modulated genes from up- (dark red) to down-regulated (dark blue) to the right of each image. The values are shown on a log_2_ scale. (b) The Y value is the log_2_-fold-changes in gene expression of the vaccinated group vs. the placebo group. The up-regulated changes are above the baseline (Y = 0, the same with placebo group), and the down-regulated changes are below the baseline. The bars represent the maximum and minimum. The mean of the fold-changes in gene expression with a 95% CI is shown as the rectangle. The line in the rectangle represents the mean of these fold changes in gene expression. The number of genes that changed after immunization is shown beside the rectangles on the image.

As shown in the neutralizing antibody analysis and the IFN-γ-specific Elispot assay, which were performed along with the microarray assay on the samples from the vaccinated subjects in the experimental cohort, a 100% antibody conversion rate was detected in the serum collected on day 28 post inoculation (p.i.) from the 20 vaccinated subjects, all of whom were anti-EV71 antibody-negative prior to the vaccination (Fig. 1a). The GMT of the neutralizing antibodies was 90.57 (95% CI 66.93–173.08) (Fig. 1a) in the vaccine cohort but only 4.00 (95% CI 4.00–4.00) in the placebo cohort (Fig. 1a). This GMT was maintained for up to 12 months after immunization, at which point the GMT was 82.32 (95% CI 65.54–135.66) (Fig. 1a). The IFN-γ-specific Elispot-based analysis of the T cells from the subjects, in which the cells were stimulated with VP1 peptides (SSIGDSVSRAL, ASSNASDESMI, STAETTLDSFF and FMSPASAYQWF) [14] from EV71, showed a distinct difference in the number of IFN-γ-producing cells between the vaccine and placebo cohorts 28 days after booster immunization (Fig. 1b). In a previous study, in which EV71-challenged neonatal rhesus monkeys were immunized with the same regimen and dose of this vaccine, the induced immune response was shown to provide effective immune protection [21], [25]. Compared with the placebo cohort, the vaccine cohort exhibited a distinctive gene expression profile associated with the immune response, including both the specific neutralizing antibody response and the cellular immune response (Fig. 1c). These results are sufficient to indicate a remarkable up-regulation of the genes associated with B cell proliferation, antibody formation and specific T cell proliferation in PBMCs, and a specific immune response was concomitantly exhibited in the vaccinated subjects (Fig. 1c).

### The Inactivated EV71 Vaccine Modulated the Expression of Genes Associated with the Immune Response

**Figure 2:**
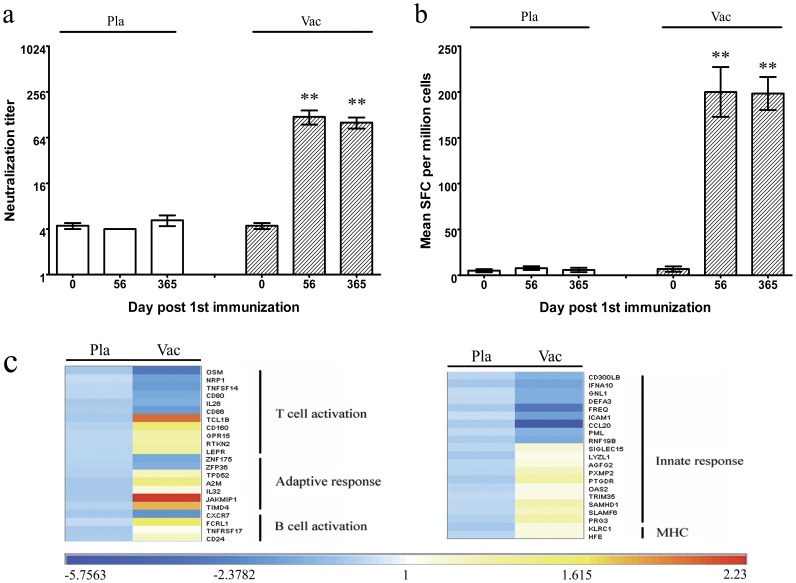
Immune responses were induced by the inactivated EV71 vaccine. The humoral (a) and cellular immune responses (b) against EV71 antigen were induced by the inactivated EV71 vaccine in volunteers (n = 20 for Vaccinated and n = 10 for Placebo). (a) The neutralizing antibodies targeting the EV71 antigen in the serum of experimental subjects. The geometric mean titers (GMTs) of neutralizing antibodies to EV71 were measured by a neutralization test as described in the Methods. **, P values of vaccinated group vs baseline value (at 0 day before primary immunization) and vs placebo group were <0.01. (b) The specific T cell response to EV71 antigenic peptides (sequences described in result section), as determined by an Elispot assay. PBMCs from the experimental subjects were incubated in the presence of 10 µM peptides, as described in the Methods section. The values are expressed as the mean±S.D. (n = 20 for Vaccinated and n = 10 for Placebo). **, P values of vaccinated group vs baseline value (at 0 day before primary immunization) and vs placebo group were <0.01. (c) The inactivated EV71 vaccination induces multiple arms of the innate and adaptive responses. Heat map of significantly modulated genes associated with immune responses against the EV71 antigen were analyzed on vaccinated cohort (Vac, n = 20) compared placebo cohort (Pla, n = 10). The listed genes were fallen under different functional categories, which included classification associated with activation of T cell, B cell, innate response adaptive response and MHC respectively. The values are shown in log_2_ scale.

The inactivated EV71 vaccine that we used in this experiment has been previously shown to induce an effective protective immune response, which was associated with gene modulation in animals [25], [31]. In this study, the EV71 vaccine was shown to induce highly variable gene expression profiles (Fig. 2), affecting 672 genes in vaccinated subjects (Tab. S3). Among these genes, 32 were directly correlated with the activity of the immune system and exhibited more than a 2-fold up-regulation (95% CI 2.32–2.77) from the baseline level, and 36 genes associated with the immune system exhibited more than a 2-fold down-regulation (95% CI 0.23–0.34) from the baseline level. As demonstrated in the functional analysis of these genes, the up-regulated genes were associated with MHC function (HFE, KLRC1); NK cell-, macrophage- and IFN-related pathways associated with innate immune responses (e.g., PRG3, SLAMF6, CCL15, SAMHD1, TRIM23, TRIM35, and OAS2); the antibody formation pathway (e.g., CD24, A2M, FCRL1, TPD52, and TNFRSF17); and specific T cell responses (e.g., RTKN2, LEPR, TIMD4, GPR15, CD160, GZMK, and JAKMIP1). The JAKMIP1 gene, which is involved in effector T cell memory, was up-regulated by more than 4-fold, revealing that the memory immune response was induced by immunization. Notably, only a few genes associated with the inflammatory response were found among the up-regulated genes, including SLURP1, which has the capacity to inhibit inflammation by modulating the release of TNF α (Tab. S3). In contrast, many genes involved in the inflammatory response (e.g., IL1α, IL1β, IL17C, CCL3, CCL23, CXCL3, CD86, CCL26, CCL4, CXCL1, NFKBIZ, IL2RA, TNFAIP2, TNFAIP6, RNF19B, SEMA7A and complement-associated C1S) were down-regulated (Tab. S3). These results suggest that immunization with the inactivated EV71 vaccine might not induce an immune-associated inflammatory response.

### The Inactivated EV71 Vaccine did not Induce an Inflammatory Response in Vaccinated Subjects

**Figure 3:**
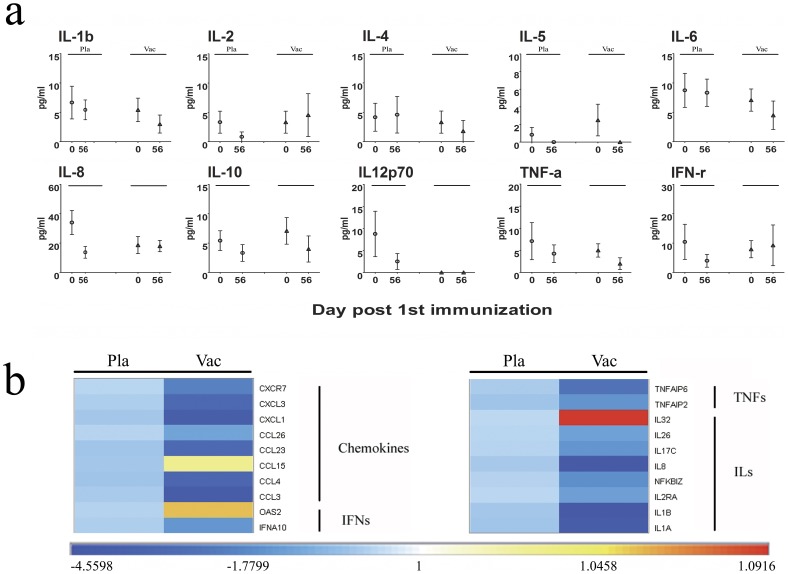
Inflammatory responses were induced by the inactivated EV71 vaccine. Measure of serum proinflammatory cytokines (a) and the modulation of the expression of genes associated with the inflammatory response (b). (a) Levels of serum proinflammatory cytokines in vaccinated and placebo subjects. All cytokine (IL-1b, IL-2, IL-4, IL-5, IL-6, IL-8, IL-10, IL12p70, TNF-α and IFN-γ) levels were measured with an R&D Systems assay kits according to the manufacturer protocol. (b) The genes modulated and associated with the inflammatory response in vaccinated group (Vac) and placebo group (Pla) were classified and fallen under different functional categories: (1) ILs group: IL1A, IL1B, IL2RA, NFKBIZ (activator of IL-6 expression), IL8, IL17C, IL26 and IL32 in the IL group; (2) TNFs group: TNFAIP2 and TNFAIP6; (3) IFNs group: IFNA10 and OAS2 (activator of IFN-γ); (4) chemokines group: CCL3, CCL4, CCL15, CCL23, CCL26, CXCL1, CXCL3 and CXCR7. The levels of gene expression were measured by microarray analysis as described in the Methods. Data are shown in log_2_ scale.

Because of the safety concerns associated with EV71 vaccines, we measured the levels of 10 pro-inflammatory cytokines in the serum taken from the vaccinated subjects and placebo group on day 28 after the booster. Similar to the placebo control cohort, the vaccine cohort did not exhibit any obvious differences in serum cytokine levels on day 0 p.i. or day 28 after the booster (Fig. 3a). This result was consistent with the inflammatory response-associated gene expression profile observed in the microarray assay (Fig. 3b). Importantly, the gene expression levels of pro-inflammatory cytokines, such as IL-1α, IL-1β, IL-6 and TNFα, and other associated genes whose expression is frequently increased in the serum of EV71-infected patients, tended to decrease in the immunized patients (Fig. 3b) However, the IFN-γ level tended to increase slightly in vaccinated subjects (Fig. 3b), and the IL-32 level clearly increased in vaccinated subjects.

### The Modulation of Genes Associated with the Immune Response was found to Correlate with the Systemic Response in Vaccinated Subjects

**Figure 4:**
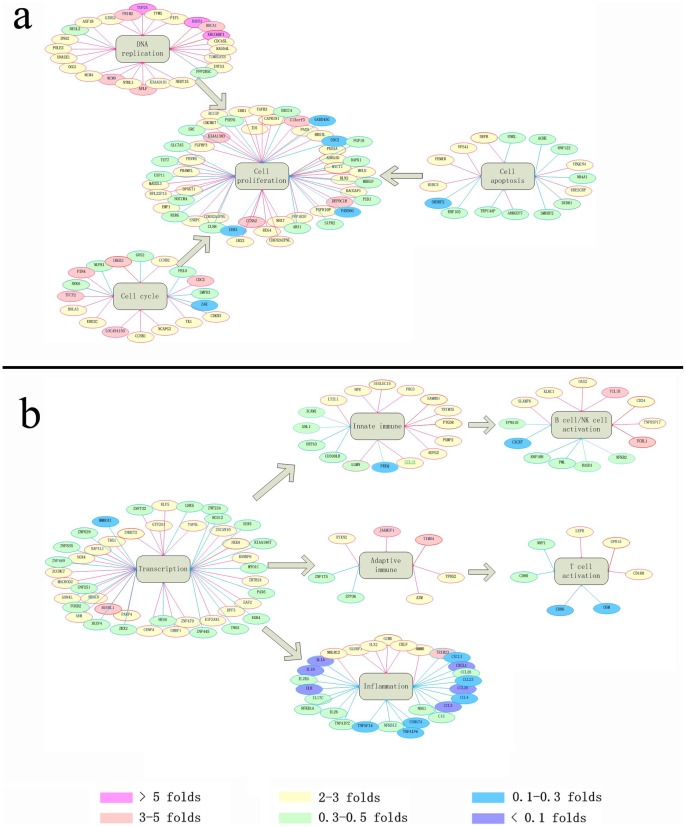
Network of differentially expressed genes after EV71 inactivated vaccine inoculation. The network represents the genes and their inferred association with cell proliferation (a) and immune responses (b) that are significantly modulated. Node colors indicate fold change of gene expression in vaccinated subjects (n = 20). The value of normal control of pre-vaccinated was 1. The color scale shows the levels of gene expression from low (green, blue and purple) to high (red, pink and yellow).

As demonstrated by the microarray results from samples of vaccinated subjects in previous studies, the specific immune response after vaccination is frequently manifested in the altered expression of genes related to cell proliferation, transcriptional regulation and signal transcription [32], [33]; these changes underlie the simultaneous modulation of the immune response [33]. The thorough characterization of this modulatory mechanism might facilitate a greater understanding of vaccine efficacy and safety. In this study, the gene expression profile induced by the inactivated EV71 vaccine in vaccinated subjects consisted of several correlated nodes (Fig. 4). First, among the up-regulated genes associated with cellular proliferation, the majority (e.g., G2NS2, PRIM2, RAD51, MCM4, MCM9, TK1, TIMELESS, TOP2A, BRCA1, PPP1R3F, SNRPC, CCNB1, BOLA3, UBE2C, TFCP2, FBXW8 and KIAA0101) are associated with cell cycle transitions from G1 phase to S phase or from G2 phase to M phase or with the promotion of DNA replication and repair (Fig. 4a). Similarly, genes such as CHEK2, MELK, BIRC5, MYCT1, RPL22P15 and BCCIP, which inhibit apoptosis, were also up-regulated (Fig. 4a). In contrast, the majority of the down-regulated genes associated with cell proliferation were correlated with promoting apoptosis (Fig. 4a). Second, among the up-regulated genes associated with transcriptional regulation, many, including TAF5L, UBQLN4, ZNF479, DPF3, ZNHIT2, GON4L, NAP1L1, CENPA, NEK9, HDAC9 and ZBTB24, were associated with the regulation of chromatin structure and epigenetic modification. Other critical components, such as KLF5, EIF2AK1, UHRF1, ZCCHC7, ZSCAN10, TRIM23, EAF2, SOX4, GTF2A1, PARP4, RUVBL1, AUH, MACROD2, TBX1, and RANBP6, promote the activation of gene transcription. The up-regulation of these genes is likely to lead to the specific and non-specific functional enhancement of immune cells and the enhancement of the subsequent immune response involving B cells, T cells and macrophages (Fig. 4b). In contrast, the majority of the down-regulated genes associated with transcriptional regulation had unclear regulatory functions or were associated with transcriptional inhibitory or pro-apoptotic functions (Fig. 4b), which may directly or indirectly contribute to the reduced expression of some pro-inflammatory cytokines (Fig. 4b). These correlations further validate the levels of the tested serum pro-inflammatory cytokines, as shown in Fig. 3.

### Confirmation of Induced Gene Modulation by Quantitative RT-PCR

**Figure 5:**
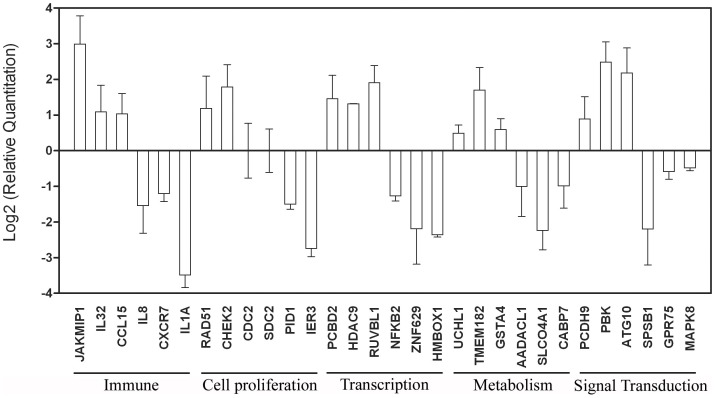
Confirmation of the genes modulated in vaccinated subjects by real-time RT-PCR. Real-time RT-PCR analyses of 30 selected genes of PBMCs from the vaccinated infants (Vac, n = 20) at day 28 after vaccine booster. The *y*-axis indicates the relative quantity of the specific mRNA in the samples compared with the pre-vaccinated samples. The results are normalised to the level of endogenous GAPDH. The error bars indicate the SD of the relative quantities.

To evaluate the expression profiles of the genes associated with the different functions detected in the microarray assay, qRT-PCR was performed on 6 genes in each of five functional classification groups (immune system-associated genes, cellular proliferation-associated genes, cellular transcriptional regulation genes, cellular metabolism-associated genes and signal transmission-associated genes), for a total of 30 genes. For each group, 3 up-regulated genes and 3 down-regulated genes were tested. The results of the qRT-PCR amplification were consistent with the microarray results (Fig. 5), suggesting that the microarray assay was reliable.

## Discussion

EV71 is generally recognized as the major causative agent of HFMD, but its pathogenesis remains poorly understood [30], [34]. A safety evaluation that considers the inflammatory response induced by and immunopathological efficacy of a vaccine is an essential step in the development of candidate vaccines. The safety of the inactivated EV71 vaccine was demonstrated in previous toxicological studies using established animal models prior to clinical trials in humans [25]; however, the potential systemic response in humans, which is presumably induced by vaccination during the generation of a specific immune response, requires further evaluation. In this paper, the characterization of the immune response induced by the EV71 vaccine is systematically described for the first time in terms of the neutralizing antibody response, the T cell response, the production of pro-inflammatory cytokines found in the serum and the modulation of gene expression in PBMCs, all of which are considered to be indicators of the specific immune response induced by vaccination. This description may provide direct evidence of the efficacy and safety of the candidate EV71 vaccine in human trials.

The inactivated EV71 vaccine was shown to induce a clear, specific immune response in terms of neutralizing antibody production in humans on day 28 after the vaccine boost (Fig. 1a). In addition, the IFN-γ-specific Elispot assay using EV71 antigens showed that a cell-mediated immune response was induced by vaccination (Fig. 1b). The microarray analysis performed on the PBMCs isolated from the whole blood of vaccinated subjects showed that the immune response induced by vaccination involved the modulation of the expression of many different genes. Interestingly, the innate immunity-associated genes, including those with functions related to identifying pathogen-derived antigens and inducing a preliminary inflammatory response, exhibited a high level of variation in the response to vaccination. These genes included KLRC1, SAMHD1, SLAMF6, CD24, HMMR, CCL15 and HFE. The genes associated with the activation of the MHC system, NK cellular activity and inflammatory responses exhibited obvious up-regulation, while the genes encoding cytokines and chemokines, such as IL-β, IL-α, CLL3, CXCL1 and CXCL3, which are usually observed in patients in the initial stage of EV71 infection, were down-regulated. However, several IFN-associated effector factors, such as OAS2, were markedly elevated. Nevertheless, some genes, including A2M, GPR15, FCRL1, RTKN2, GZMK, TPD52, TNFRSF17, TCL1B, TRIM23, TRIM35, and JAKMIP1, which are involved in promoting the humoral immune response and T cell activation, exhibited obvious up-regulation. Collectively, the up-regulation of the genes directly associated with immune activation is presumed to be correlated with the integrated mobilization of the corresponding immune cell populations that make up the PBMCs. As shown in the expression analysis of genes associated with cell proliferation, the DNA replication-associated genes, such as GINS2, TYMS, RAD51, PRIM2, ASF1B, TOP2A, MCM4 and MCM9, tended to be clearly up-regulated. PRIM and RAD51 were up-regulated by 4-fold and 5-fold, respectively, and the up-regulation of the TOP2A gene, which encodes an enzyme that is critical for DNA replication, exceeded 6-fold (Tab. S3). Additionally, several cell cycle genes, such as CDC45L, BRCA1, PPP1R3F, SNRPC, CDKN3, and CCNB1, which are responsible for promoting the G1 to S phase and G2 to M phase transitions, were up-regulated, whereas pro-apoptosis genes, such as NUPR1, RNF122, CABLES1, NR4A1, 2AK, and DIDD1, tended to be down-regulated (Fig. 4a). Furthermore, the results suggested that the immune cells were mobilized in an integrated manner involving the up-regulation of the genes associated with chromatin structure remodeling and epigenetic modification, such as UBQLN4, POLE2, DPE3, CEWPA, ZNF479, and GON4L, and genes related to essential regulatory functions in immune cell proliferation, such as SOX4, EAF2, ZSCAN10, TBX1, and GTF2A1. The up-regulation of these effector genes is likely to contribute to the integrated functional activation of the immune system. No significant changes in gene regulation were observed in the placebo cohort, indicating that the systemic response and its correlated specific immune response were induced by the EV71 vaccine.

Another characteristic of the systemic immune response was the down-regulation of the expression of genes encoding pro-inflammatory cytokines and chemokines. This down-regulation was supported by the levels of serum pro-inflammatory factors (Fig. 3). The gene expression levels of adhesion factors, such as ICAM and CD300 LB, which have immunopathological importance in the inflammatory response, were down-regulated relative to the levels found in the placebo group (Tab. S3) [35], [36]. In clinical studies of EV71-infected HFMD patients, especially fatal cases, it has been demonstrated that the levels of pro-inflammatory cytokines, such as IFN-γ, IL-6 and TNFα, in the blood and cerebrospinal fluid are increased [22], [37]. These increased cytokine levels may pose a safety concern for the use of the EV71 vaccine in humans. The data obtained in this work indicate that no inflammatory reaction was involved in the immune response induced by the vaccine. Although the cytokine levels in the serum in this study might have reverted to normal by 28 days after vaccination, the fact that the increasing antibody titers and the activation of T cells were not associated with changes in the levels of inflammatory factors at this time further supports the safety of this vaccine. Generally, it can be hypothesized that an EV71 vaccine composed of the purified antigen from an inactivated virus generates an immune response and that the inactivated virus might not induce a strong innate immune response. Certainly, further studies should explore the responses of the vaccinated population to viral challenge. As demonstrated in our previous study of EV71 vaccine protection in neonatal rhesus monkeys, the levels of some cytokines in monkeys immunized with high-dose EV71 vaccine tended to increase upon virus challenge [21].

## Supporting Information

Table S1 Basic characteristics of 30 participants in this study.
(DOC)

Table S2 Sequences of primers for real-time RT-PCR amplification of 30 selected genes.
(DOC)

Table S3 The genes modulated after administration of EV71 inactivated vaccine (human diploid cells).
(DOC)
