# Supplementary material for: Study of the Integrated Immune Response Induced by an Inactivated EV71 Vaccine
Source: PLoS One. 2013 Jan 23;8(1):e54451. doi: 10.1371/journal.pone.0054451 (PMC3553120; doi:10.1371/journal.pone.0054451)
Supplement: Table S1 — Basic characteristics of 30 participants in this study. (DOC) [file pone.0054451.s001.doc]

**Table S1** Basic characteristics of 30 participants in this study

|  |  | **Vaccine group** |  | **Placebo group** |
| --- | --- | --- | --- | --- |
|  |  | 100U (320EU) |  | 0U (0EU) |
| Volunteers ^a^ |  | 20 |  | 10 |
| Male to female ratio ^b^ |  | 1.22 (11/9) |  | 1.00 (5/5) |
| Age ^c^ |  | 9.57±1.56 |  | 9.12±1.32 |
| Weight ^c^ |  | 9.74±2.95 |  | 9.81±2.70 |
| Anti-EV71 antibody |  |  |  |  |
| Prevalence GMT ^d^ |  | 4.40 (3.56-5.24) |  | 4.40 (3.50-5.30) |

a. The number of randomized participants who received two dose of vaccine or placebo based on the clinical trail protocol.

b. The number of male person rated to the number of female person.

c. The results are shown as Mean±SD. Age: months-old; weight: kg.

d. The GMT of volunteers before immunization
