# Supplementary material for: Study of the Integrated Immune Response Induced by an Inactivated EV71 Vaccine
Source: PLoS One. 2013 Jan 23;8(1):e54451. doi: 10.1371/journal.pone.0054451 (PMC3553120; doi:10.1371/journal.pone.0054451)
Supplement: Table S2 — Sequences of primers for real-time RT-PCR amplification of 30 selected genes. (DOC) [file pone.0054451.s002.doc]

**Table S2** Sequences of primers for real-time RT-PCR amplification of 30 selected genes.

| **Classical** | **Gene name** | **Primer sequence (5’→3’)** | | **Target size (bp)** |
| --- | --- | --- | --- | --- |
|  |  | **Sense** | **Anti-sense** |  |
| **Immune response** | JAKMIP1 | TGCAGAGTATCCAGAGGATGGA | GCTGACAGCTTTTCTTTCATTTCC | 79 |
|  | IL32 | TGGTGGCCCTGGTCCAT | AGAGAGCAGCAGAAACTCTGGAA | 64 |
|  | CCL15 | TGCTGCACCTCCTACATCTCA | CGCTGCTCGTTTCAAAATAACTT | 70 |
|  | IL8 | TCTTGGCAGCCTTCCTGATT | TCTTTAGCACTCCTTGGCAAAAC | 67 |
|  | CXCR7 | ACACGCACTGCTACATCTTGAAC | AGACTGGGATGGTGAGGACAA | 66 |
|  | IL1A | CCCAAAACCATCACAGGTAGTGA | GTGAAATAGTTCTTAGTGCCGTGAGT | 74 |
|  |  |  |  |  |
| **Cell proliferation** | RAD51 | GAGCTTTCAGCCAGGCAGAT | TCATCAGCGAGTCGCAGAAG | 65 |
|  | CHEK2 | GGGCAGAAGTCTCAGAGAAAGCT | AAAACGTGCCTTTGGATCCA | 71 |
|  | CDC2 | AGACACAAAACTACAGGTCAAGTGGTA | TTCCCGAATTGCAGTACTAGGAA | 90 |
|  | SDC2 | GGCTTTCTCTTTGCAATTTTTCTT | CATAGCTTCCTTCATCCTTCTTTCTC | 73 |
|  | PID1 | CGCTAGCCCGAGAGGATGT | AACTTGGAATGGCCGGATTT | 63 |
|  | IER3 | AACCGAACCCAGCCAAAAG | CCATCAGGATCTGGCAGAAGA | 66 |
|  |  |  |  |  |
| **Transcription** | PCBD2 | TTTAATCAGGCATTTGGCTTTATG | CCATTCTGGGTGATGATTCATCT | 72 |
|  | HDAC9 | GACACCATTTGGAATGAGCTACAC | GCTCGATGACACAGCCAACA | 67 |
|  | RUVBL1 | CGCGCCCTGGAGTCTTC | CACAGTTGCC TCGGTTGGAT | 61 |
|  | NFKB2 | GGGATCTGCGCCGTTTCT | ACATGCAGGACACCCAGGTT | 71 |
|  | ZNF629 | CGACGAGGGCATCTTCATG | AGGCCGTCTGCATTTTTGTAG | 72 |
|  | HMBOX1 | CCCCATCTCATTAGCTGTGGAA | CCTTGTCGGGCCAATGC | 66 |
|  |  |  |  |  |
| **Metabolism** | UCHL1 | AACTTGATGGACGAATGCCTTT | TCAGCAGGGTGTCCTCTGAAC | 63 |
|  | TMEM182 | CATCGCAAGCTTTTTGATCATC | CCTCCCCCAGCTTTGTAGAGA | 69 |
|  | GSTA4 | GCAGCAAGGCCCAAGCT | AACCCATCTCACGGACTCCAT | 63 |
|  | AADACL1 | CGCAGCGTCGTTTATATCCA | GGTGACCAGGACGCACTTG | 65 |
|  | SLCO4A1 | ACCTACCTGGATGAGAACGTCAA | CGCTGTGTAGAAGATGGCAATG | 66 |
|  | CABP7 | GAAGTGCGACATGCAGAAGCT | GCTCGCAGAAGGTGTCGTAGA | 68 |
|  |  |  |  |  |
| **Signal Transduction** | PCDH9 | CGCCGGTGCCATGGT | GCTGCTTTGAACCTTGATGCA | 81 |
|  | PBK | GACTGACCCTGAGGCTTGTTACA | ACACCATTCTCCTCCACAGCTT | 72 |
|  | ATG10 | TCCCATGGAGGAGGCTTTC | GGACGCTGCTGCAGTTTCA | 67 |
|  | SPSB1 | CGACCGATCGCTCAATGTC | CCGGATGCCGGTGAAAG | 62 |
|  | GPR75 | AAAGCCGTGGTCACCTGTGT | AAGGAAATCCCCAGTGGAAGA | 71 |
|  | MAPK8 | ACAGAGCACCCGAGGTCATC | TGCACCCCACAGACCATAAA | 69 |
