# Supplementary material for: Study of the Integrated Immune Response Induced by an Inactivated EV71 Vaccine
Source: PLoS One. 2013 Jan 23;8(1):e54451. doi: 10.1371/journal.pone.0054451 (PMC3553120; doi:10.1371/journal.pone.0054451)
Supplement: Table S3 — The genes modulated after administration of EV71 inactivated vaccine (human diploid cells). (DOC) [file pone.0054451.s003.doc]

**Table S3** The genes modulated after administration of EV71 inactivated vaccine (human diploid cells)

| **Gene levels** | **Classes** | **Subclasses** | **Protein name** | **Folds** | **Functions** |
| --- | --- | --- | --- | --- | --- |
| **Up-regulated** | |  |  |  |  |
|  | **Immune response** | -ILs | IL32 | 2.1311 | strongly associated with TNFα, IL-1β, and IL-18; play a role in human RA and may be a novel target in autoimmune diseases |
|  |  |  | A2M | 2.8506 | a wide array of cytokines and proteases by an unusual mechanism; high concentrations in the plasma and inflammatory fluids; associate with TNF-α, IL-1ß, IL-2, IL-6, IL-8 |
|  |  | -IFNs | OAS2 | 2.0792 | implicated in the mechanism of the antiviral action of interferon; associate with IFNs |
|  |  | -Chemokines | CCL15 | 2.0426 | induces recruitment neutrophils, monocytes, and lymphocytes |
|  |  | -Others | HFE | 2.1395 | similar to MHC class I-type proteins and associates with beta2-microglobulin (beta2M) |
|  |  |  | KLRC1 | 2.1053 | recognition of the MHC class I HLA-E molecules in NK cells |
|  |  |  | PRG3 | 2.4302 | paralog of major basic protein (MBP1) and, similar to MBP1, is cytotoxic and cytostimulatory in vitro |
|  |  |  | SLAMF6 | 2.2797 | expressed on Natural killer (NK), T, and B lymphocytes; coreceptor in the process of NK cell activation |
|  |  |  | SAMHD1 | 2.4231 | regulation of the innate immune response |
|  |  |  | TRIM35 | 2.0984 | a part of the innate immune system to counter intracellular pathogens |
|  |  |  | TRIM23 | 3.5018 | TRIM23-mediated ubiquitin conjugation to NEMO (NF-κB essential modulator ) is essential for TLR3- and RIG-I/MDA5-mediated antiviral innate and inflammatory responses |
|  |  |  | PTGDR | 2.4634 | G-protein-coupled receptor; affects other cells in the allergic immune response |
|  |  |  | PXMP2 | 2.3199 | an integral membrane protein; involved in the host response against CVB3 (coxsackievirus B3) |
|  |  |  | LEPR | 2.5234 | involved in the regulation of fat metabolism; immune responses toward T helper-1 (Th1) cytokine production |
|  |  |  | TIMD4 | 3.436 | expressed on macrophages and dendritic cells; regulate adaptive immunity |
|  |  |  | RTKN2 | 2.4664 | play a role in activation signalling, lymphocyte division, and differentiation; play a role in the function of the immune system |
|  |  |  | GZMK | 2.0919 | proinflammatory activity; activates pro-interleukin-1β |
|  |  |  | GPR15 | 2.4306 | expressed on CD4+ T lymphocytes, a major target cell for SIV infection in vivo, and on alveolar macrophages |
|  |  |  | CD160 | 2.9182 | a glycosylphosphatidylinositol-anchored member of the immunoglobulin superfamily, is expressed on both cytolytic lymphocytes and some unstimulated CD4+ T cells |
|  |  |  | JAKMIP1 | 4.6915 | highlight a role in the maintenance of a controlled cytotoxic immune response in humans |
|  |  |  | AGFG2 | 2.1213 | a member of the HIV-1 Rev binding protein (HRB) family |
|  |  |  | TCL1B | 4.0429 | expressed mainly in CD4-/CD8- immature T-cells, pre B-cells and virgin B-cells |
|  |  |  | HMMR | 2.6041 | a hyaluronan receptor；involved with inflammation |
|  |  |  | CD24 | 2.2912 | expressed on mature granulocytes and in many B cells |
|  |  |  | TNFRSF17 | 2.0522 | expressed in mature B lymphocytes, and may be important for the development of B cells |
|  |  |  | FCRL1 | 3.0968 | play a role in regulating cellular differentiation and modulating the initiation and termination of B-cell responses. |
|  |  |  | TPD52 | 2.2922 | a role in cell proliferation, apoptosis, vesicle trafficking, tumorigenesis, and metastasis；inducing an immune response |
|  |  |  | LYZL1 | 2.0732 | response to an immune challenge |
|  |  |  | CKLF | 2.4038 | novel cytokine; have important roles in inflammation and in the regeneration of skeletal muscle. |
|  |  |  | SLURP1 | 2.8654 | controls TNF-α release in dermal macrophages and in keratinocytes by activation of nAChRs and thus reduced inflammation. |
|  |  |  | NHLRC2 | 2.0324 | associated with inflammationary response |
|  |  |  | SIGLEC15 | 2.1893 | be localized inside macrophages/dendritic cells; involved in the immune surveillance of tumors. |
|  |  |  |  |  |  |
|  | **Transportation and metabolism** | - Energy metabolism and oxidation | CUBN | 2.7377 | a receptor for intrinsic factor-vitamin B12 complexes |
|  |  |  | GGNBP1 | 2.121 | a mitochondrial protein; involved in spermatogenesis by modifying mitochondrial dynamics and morphology |
|  |  |  | SLC25A10 | 2.5066 | catalyzes the transport of dicarboxylates across the mitochondrial membrane |
|  |  |  | AGMAT | 2.0189 | hydrolyzes agmatine to putrescine and urea; Its expression in human liver is induced during hepatitis B virus infection |
|  |  |  | GALK2 | 2.0912 | galactokinase |
|  |  |  | LOXL2 | 2.1464 | implicated in cross-link formation in stromal collagens and elastin, cell motility and tumor progression; extracellular matrix metalloenzyme |
|  |  |  | ULK2 | 2.0285 | required in the autophagy response; glucose metabolism |
|  |  |  | HDHD2 | 2.7317 | involvement in sugar phosphate phosphotransferase activity |
|  |  |  | CAPN8 | 2.103 | modulator protease |
|  |  |  | FMO5 | 2.0849 | FMO5 has been reported to S-oxygenate thioethers with a proximal carboxylic acid |
|  |  |  | PIGW | 3.8824 | histidine kinase；phosphatidylinositol glycan anchor biosynthesis |
|  |  |  | MAN2C1 | 2.3622 | role in the catabolism of cytosolic free oligomannosides |
|  |  |  | DGKE | 2.9821 | an enzyme that is responsible for controlling the cellular level of DG by converting DG to phosphatidic acid (PA) |
|  |  |  | SCLY | 2.0604 | specifically catalyzes the decomposition of l-selenocysteine tol-alanine and elemental selenium |
|  |  | -Lipid metabolism | B3GALT2 | 2.1702 | type II membrane-bound glycoproteins; beta-1,3-galactosyltransferase |
|  |  |  | LPCAT4 | 2.7833 | catalyze the conversion of lysophosphatidic acid (LPA) to phosphatidic acid (PA) |
|  |  |  | PIP4K2B | 2.0342 | a lipid kinase；interacts with p55 TNF receptor |
|  |  |  | CYP4V2 | 2.1882 | implicated in the metabolism of fatty acid |
|  |  |  | PECR | 2.651 | an enzyme with an unknown function but proposed to play a role in fatty acid chain elongation |
|  |  |  | FABP5 | 2.0688 | an important role in the primary protection against the cellular toxicity of cholesterol, free fatty acids, and/or lipid oxidants |
|  |  | - Signal transduction | OR10P1 | 2.199 | G-protein-coupled receptors (GPCR); initiate a neuronal response that triggers the perception of a smell |
|  |  |  | TBC1D24 | 2.9203 | interact with GTPases; involved in the regulation of membrane trafficking |
|  |  |  | KRT38 | 2.2167 | a type I hair keratin; a member of the keratin gene family |
|  |  |  | MYLIP | 3.3752 | myosin regulatory light chain interacting protein |
|  |  |  | NCDN | 5.671 | expressed in the hindbrain, spinal cord, the diencephalon and telencephalon |
|  |  |  | ATP2B4 | 2.188 | plays a critical role in intracellular calcium homeostasis |
|  |  |  | BLZF1 | 3.0343 | triggers replication of the Epstein–Barr virus (EBV) |
|  |  |  | DGKH | 2.1357 | regulating the intracellular concentrations of diacylglycerol and phosphatidic acid; regulatory component of the Ras/B-Raf/C-Raf/MEK/ERK signaling cascade |
|  |  |  | MMP19 | 2.3061 | involved in processes such as neovascularization and angiogenesis. |
|  |  | - Protein transportation and metabolism | FBLN5 | 2.0904 | promotes adhesion of endothelial cells; a role in vascular development and remodeling; pathologic alterations affecting peripheral nerves |
|  |  |  | IFT80 | 2.0395 | part of the intraflagellar transport complex B; function of motile and sensory cilia. |
|  |  |  | ALS2CR4 | 2.0433 | involved in membrane transport between the photoreceptor inner and outer segments; maintaining the structural integrity of the outer segment |
|  |  |  | SPATA7 | 2.3628 | involved in protein transport |
|  |  |  | MFAP5 | 2.1121 | critical extracellular matrix gene transcripts |
|  |  |  | PPAPDC1B | 2.2381 | involved in vesicular protein transport and membrane trafficking between the Golgi apparatus and the cell surface; associated with chlathrin-coated pits and increased in the tumor cells |
|  |  |  | NUTF2 | 2.2171 | import of proteins into the nucleus through the nuclear pore complex |
|  |  |  | ATG7 | 2.4993 | exclusively associated with autophagy, is required for membrane trafficking and turnover in the axons |
|  |  |  | DPY19L1 | 2.1148 | a gene expressed strongly in cortical glutamatergic neurons and as a regulator of radial neuronal migration; be associated with various diseases |
|  |  |  | PLEKHA8 | 2.7421 | is crucial for molecular transport |
|  |  |  | M6PR | 2.2111 | mediate the transport of newly synthesized lysosomal enzymes with an M6P recognition motif from the TGN to lysosomes |
|  |  |  | RNF175 | 2.0465 | involved in metal ion binding |
|  |  |  | TMCO6 | 2.2248 | collagenous transmembrane proteins |
|  |  |  | RAB15 | 2.1177 | specifically expressed in brain and localized to synaptic vesicles; play a role in neurotransmitter release by regulating membrane flow in the nerve terminal |
|  |  |  | OCLM | 2.0246 | function of the trabecular meshwork and also in the development of primary open-angle glaucoma |
|  |  |  | OR5A1 | 2.1533 | an olfactory receptor belonging to the largest multigene family, expressed in a wide variety of metazoan species |
|  |  |  | EPDR1 | 3.9225 | a transmembrane protein |
|  |  |  | ATP5G3 | 2.4949 | mitochondrial ATP synthase subunit 9；H+ transporting |
|  |  |  | MFSD3 | 4.4508 | membrane transporters |
|  |  |  | FNDC3A | 5.0548 | mediating spermatid–Sertoli adhesion during mouse spermatogenesis |
|  |  |  | SFXN2 | 3.2076 | a mitochondrial transmembrane protein; important in the differentiation of pancreatic β-cells as a channel or a carrier molecule and be related to the regeneration of pancreatic endocrine cells |
|  |  |  | LOC389842 | 2.1583 | important for Ran function in protein import |
|  |  |  | VNN1 | 2.722 | participate in hematopoietic cell trafficking and to possess pantetheinase activity which may play a role in oxidative-stress response |
|  |  |  | OIP5 | 3.3788 | involved in gonococcal adhesion to and invasion of human epithelial cell |
|  |  |  | RER1 | 2.7299 | containing four putative membrane spanning domains. |
|  |  |  | NPPB | 2.3994 | a mentoer of the novel class of Cl– channel blockers related to diphenylamine-2-carboxylate |
|  |  |  | KCNF1 | 2.5265 | involved in determining the resting potential of cell membranes |
|  |  |  | NIPA1 | 2.1014 | highly expressed in neuronal tissues and encodes a putative membrane transporter or receptor |
|  |  |  | ABCG2 | 2.5337 | transporters have an important role in maintaining the barrier function of sanctuary site tissues |
|  |  |  | STON1 | 2.1926 | related to components of the endocytic machinery |
|  |  |  | HIGD1A | 2.8102 | arylhydrocarbon receptor nuclear translocator |
|  |  |  | SLC43A1 | 2.3434 | important role in the efflux transport |
|  |  |  | ANXA2P3 | 3.6486 | stimulate tissue-type plasminogen activator (t-PA)–dependent plasminogen activation by serving as a coreceptor for t-PA and plasminogen |
|  |  |  | AP1S1 | 3.2132 | involved in the organisation and transport of many other proteins within the cell |
|  |  |  | MMRN2 | 4.6605 | supports cell adhesion and binds to the integrins αIIbβ3 and αvβ3, which are targets of drug treatments for acute arterial thrombosis |
|  |  |  | ALDH18A1 | 2.1752 | ATP- and NADPH-dependent mitochondrial enzyme |
|  |  |  | HIBCH | 2.1351 | hydrolysis of both HIBYL-CoA and beta-hydroxypropionyl-CoA |
|  |  |  | POP5 | 2.0838 | Subunit of the Human RNase MRP and RNase P Endoribonucleases |
|  |  |  | COQ2 | 2.7412 | an enzyme that functions in the final steps in the biosynthesis of CoQ |
|  |  |  | ADAL | 2.3666 | a catalytic zinc ion |
|  |  |  | FIG4 | 2.4589 | an inositol phosphatase; regulation of various phosphoinositides, and affects diverse cellular functions such as actin cytoskeleton organization, Golgi function, and maintenance of vacuole morphology |
|  |  |  | ASRGL1 | 2.0206 | an enzyme responsible for the conversion of alanine to pyruvate and glycine |
|  |  |  | LRTOMT | 2.7067 | a candidate catechol-O-methyltransferase |
|  |  |  | MBOAT2 | 2.8625 | a lysophosphatidylserine (lyso-PS) acyltransferase with preference for oleoyl-CoA; play a critical role in the regulated synthesis of LTB4 in the human neutrophil |
|  |  |  | PRMT7 | 2.2563 | a Type II methyltransferase |
|  |  |  | DPP4 | 2.8554 | serine protease; responsible for hyperglycaemia, lipid metabolism and preservation of renal function |
|  |  |  | ABHD1 | 2.0047 | an enzymatic function such as an esterase, lipase or thioesterase |
|  |  |  | LSS | 2.1714 | Partial inhibition of OSC should reduce synthesis of lanosterol and subsequent sterols; a unique target for a cholesterol lowering drug |
|  |  |  | GCAT | 2.3823 | catalyze manifold reactions in the metabolism of amino acids |
|  |  |  | GSTA4 | 4.0993 | catalyzes the detoxification of HNE and related lipid peroxides by conjugation to GSH; involved in detoxification and protection of cells against chemical and oxidative stress |
|  |  |  | HSD17B6 | 2.7073 | associated with several metabolic phenotypes |
|  |  |  | DHFRL1 | 2.9384 | the only enzyme capable of the reduction of dihydrofolate to tetrahydrofolate |
|  |  |  | PGP | 2.1312 | a key enzyme of photorespiration in photosynthetic organisms |
|  |  |  | ANKMY1 | 3.0432 | have ubiquitin ligase and/or histone methyltransferase activity |
|  |  |  | CBR4 | 2.0285 | NADPH-dependent carbonyl reductase from human brain (formerly designated as aldehyde reductase 1; reduces a number of biologically and pharmacologically active carbonyl compounds |
|  |  |  | CBS | 2.6968 | a known pyridoxal 5'-phosphate (PLP) enzyme; catalyzed the first committed step of transsulfuration |
|  |  |  | MOSC1 | 2.4172 | a novel class of molybdoenzyme; play a role in protection against mutagenic base analogues and related carcinogens in humans |
|  |  |  | PYCR1 | 2.4196 | involved in the proline metabolic pathway that catalyzes the obligatory and final step in de novo proline synthesis |
|  |  |  | FAR2 | 2.2025 | a novel neural member of the Ig superfamily, which is related to F11/F3/contactin and axonin-1/TAG-1 |
|  |  |  | SERPINI1 | 2.3872 | brain disease-related genes; the protease inhibitor |
|  |  |  | NUDT3 | 2.0435 | involved in nucleoside metabolism, playing a role in the removal of some toxic nucleotide metabolites |
|  |  |  |  |  |  |
|  | **Cell proliferation** | - DNA replication | GINS2 | 2.4515 | the initiation of DNA replication; DNA damage, and impairing cell proliferation on inhibition |
|  |  |  | PRIM2 | 4.2227 | replication of DNA; synthesizes small RNA primers |
|  |  |  | TYMS | 2.1632 | DNA replication and repair |
|  |  |  | RAD51 | 5.0848 | involved in the homologous recombination and repair of DNA |
|  |  |  | RAD54L | 2.7261 | involved in the homologous recombination and repair of DNA; induces a DNA topological change |
|  |  |  | POLE2 | 2.2322 | DNA replication and repair; interacts with SAP18 and recruit histone deacetylase (HDAC) |
|  |  |  | DNASE1 | 2.2777 | DNase1 gene |
|  |  |  | MCM4 | 2.3411 | DNA replication; as a replicative helicase at replication forks |
|  |  |  | MCM9 | 3.4371 | essential for pre-RC formation and DNA replication |
|  |  |  | APLF | 3.5869 | a novel component of the cellular response to chromosomal DNA single- and double-strand breaks |
|  |  |  | TIMELESS | 2.2471 | important for replication checkpoint and normal DNA replication processes, functions as a replication fork stabilizer |
|  |  |  | CDC45L | 2.3584 | a possible role of the protein in the initiation of DNA replication. |
|  |  |  | ASF1B | 2.0968 | regulates the progression of cellular DNA replication forks via chromatin reorganization |
|  |  |  | TOP2A | 6.0129 | a key enzyme in DNA replication and a molecular target for many important anticancer drugs |
|  |  |  | PIF1 | 2.0018 | a single-stranded (ss) DNA-dependent ATPase and a DNA helicase which unwinds partially DNA duplexes in a 5' to 3' direction with respect to the ss DNA on which it binds first |
|  |  |  | BRCA1 | 3.1371 | (HR)-mediated DNA repair; expressed during the S and G2 phases of the cell cycle |
|  |  |  | XRCC6BP1 | 6.0959 | part of the DNA-dependent protein kinase complex involved in double-strand break repair |
|  |  |  | NUDT15 | 2.1408 | a mutagenic substrate for DNA synthesis, in the nucleotide pool, thereby preventing DNA replication errors |
|  |  |  | INTS3 | 2.3853 | required for an effective DNA damage response |
|  |  |  | KIAA0101 | 2.257 | involved in DNA replication and damage repair; involved in the regulation of DNA repair, apoptosis and cell cycle progression |
|  |  |  | NTHL1 | 2.5329 | a DNA glycosylase/AP lyase involved in base excision repair |
|  |  |  | OGG1 | 2.2859 | the protection of the genome from the mutagenic effects of the oxidatively damaged purines |
|  |  |  | DNA2 | 2.2214 | maintenance of chromosomal integrity, such as repair or other related process |
|  |  | - Translation | HJURP | 2.4194 | a CENP-A-specific histone chaperone |
|  |  |  | RDM1 | 4.6394 | a nucleic-acid binding domain of the RNA recognition motif (RRM) type; recognize distortions of the double helix |
|  |  |  | SMUG1 | 2.0153 | converge upon a series of downstream repair proteins |
|  |  |  | STRBP | 2.1446 | Belong to RNA-binding proteins (RBPs) |
|  |  |  | STT3A | 2.4607 | play complementary roles in the cotranslational and posttranslational N-glycosylation of proteins |
|  |  |  | SFRS2B | 2.506 | alternative splicing; roles in RNA processing including control of export, translation, stability, and constitutive and alternative splicing |
|  |  |  | TTLL1 | 3.0145 | posttranslational polyglutamylation of tubulin in axonemal microtubules within cilia and flagella;catalytic subunit of a tubulin glutamylase |
|  |  |  | TTLL12 | 3.4996 | catalyze posttranslational modification of tubulins; effects on the cytoskeleton, tubulin modification and chromosome number stability |
|  |  |  | ESRP2 | 2.164 | Epithelial cell-type-specific splicing proteins, ESRP1 and ESRP2, that are required for the expression of epithelial FGFR2-IIIb |
|  |  |  | PAIP2B | 3.6464 | inhibit translation of capped and polyadenylated mRNAs |
|  |  |  | DNAJC21 | 2.433 | required for one of the final steps in formation of the 60S ribosomal subunit |
|  |  | - cell cycle | PIN4 | 3.1362 | a role in the cell cycle, chromatin remodeling, and/or ribosome biogenesis; accumulate at chromosomes during mitosis |
|  |  |  | CHEK2 | 4.2972 | key mediators of cellular responses to DNA damage; control cell cycle and DNA repair |
|  |  |  | CCNB2 | 2.9773 | cell cycle regulatory |
|  |  |  | CDC2 | 3.0731 | prototypical cyclin-dependent kinase that promotes G2-M phase transition in the cell cycle |
|  |  |  | CDKN3 | 2.671 | negative cell cycle regulators of regulation of the G1- to S-cell cycle phase transition |
|  |  |  | NCAPG2 | 2.3414 | promotes mitotic chromosome condensation, and its supercoiling activity increases during mitosis by Cdc2 phosphorylation |
|  |  |  | CCNB1 | 2.3621 | essential for the G2/M transition of the cell cycle |
|  |  |  | LOC494150 | 3.3644 | as normal cell cycle regulation, replicative senescence, cellular immortalization, and the development of sporadic breast tumors |
|  |  |  |  |  |  |
|  |  |  | UBE2C | 2.0272 | the cell-cycle G2–M phase gene; driving M-phase cell-cycle progression by inactivating the M phase checkpoint or increasing the pool of active APC/C |
|  |  |  | BOLA3 | 2.1017 | involved in cell proliferation or cell-cycle regulation |
|  |  |  | TFCP2 | 3.8901 | plays a critical role in progression from G1 to S phase of the cell cycle |
|  |  |  | TK1 | 2.0809 | a cell cycle-regulated enzyme; Its activity fluctuates with DNA synthesis |
|  |  | - Ubiquitin-proteosome | FEM1B | 2.0042 | a role in apoptosis |
|  |  |  | VPS41 | 2.777 | involved in lysosomal trafficking; block downstream events in the apoptotic cascade |
|  |  |  | DFFB | 2.2282 | induce apoptotic chromatin condensation |
|  |  |  | FBXL16 | 2.6574 | interactions with the E3 ubiquitin ligase complex |
|  |  |  | FBXO4 | 2.741 | ubiquitin protein ligase complex; phosphorylation-dependent ubiquitination; tumor suppressor properties; a negative regulator of cyclin D1 |
|  |  |  | UBQLN4 | 2.5897 | involved in proteasome-mediated degradation of proteins and interacts with ataxin-1 |
|  |  |  | USP36 | 2.2784 | deubiquitylating enzyme |
|  |  |  | UBE2CBP | 3.049 | ubiquitin-conjugating enzyme E2C binding protein |
|  |  |  | HERC4 | 2.6585 | involving an E1 ubiquitin-activating enzyme |
|  |  |  | ASB9 | 2.6292 | mediates ubiquitination and proteasomal degradation of CKB in the cells |
|  |  |  | RNF170 | 3.2224 | plays an essential role in IP3 receptor processing via the ubiquitin-proteasome pathway |
|  |  |  | BRI3BP | 4.0881 | pro-apoptotic property and can potentiate drug-induced apoptosis; involved in the structural dynamics of the ER and affects mitochondrial viability |
|  |  |  | USP13 | 2.1011 | a SUMO modifying enzyme; catalyzing hydrolysis of various Ub chains |
|  |  |  | UCHL1 | 3.9373 | participated in postthymic proliferation |
|  |  |  | DDX25 | 2.4177 | a testis-specific member of the DEAD-box family of RNA helicases; associated with germ cell development |
|  |  |  | BIRC5 | 2.0249 | a protein in the intrinsic apoptotic pathway that interacts with XIAP and DIABLO leading to caspase-3 and caspase-9 inactivation |
|  |  |  | APAF1 | 2.4926 | crucial for PCD (programmed cell death ) in the developing brain during the embryogenesis; regulating the activation of Casp2, Casp3, and Casp8 |
|  |  |  | TXNRD2 | 2.2421 | involved in apoptosis regulation |
|  |  | - Signal transduction | HBS1L | 2.3118 | GTP-binding elongation factor; controlling fetal hemoglobin level |
|  |  |  | TGFB3 | 2.4554 | involved in embryogenesis and cell differentiation; implicated in the control of lung branching morphogenesis |
|  |  |  | ID1 | 2.0219 | inhibit the DNA binding and transcriptional activation ability of basic HLH proteins; a key regulator for the proliferation and differentiation of cells |
|  |  |  | PAX5 | 2.1968 | regulators in early development; proliferative activity |
|  |  |  | ADRA1D | 2.4573 | G protein-coupled receptor; activate mitogenic responses and regulate growth and proliferation of many cells |
|  |  |  | MELK | 2.356 | an embryonic and neural stem cell marker; cell survival, cell proliferation, and apoptosis |
|  |  |  | RACGAP1 | 2.1862 | GTPase-activating protein; regulatory role in initiation of cytokinesis, controlling cell growth and differentiation |
|  |  |  | FGFR1OP | 2.0591 | role in normal proliferation and differentiation of the erythroid lineage; involved in growth and proliferation |
|  |  |  | PPP1R3F | 2.2658 | one of several type-1 protein phosphatase (PP1) regulatory subunits |
|  |  |  | NEK7 | 2.1585 | controls initiation of mitosis; participate in a mitotic cascade |
|  |  |  | CCNA2 | 4.6539 | growth in both cell lines |
|  |  |  | SNRPC | 2.7108 | proliferation-associated in B cell |
|  |  |  | DPAGT1 | 2.5313 | is associated with cell proliferation and nascent adherens junctions |
|  |  |  | PRAMEL | 2.1068 | expressed up to the epiblast stage of embryonic development and in undifferentiated mESCs |
|  |  |  | FGFBP3 | 2.385 | fibroblast growth factor binding protein |
|  |  |  | KIAA1383 | 3.1166 | cell growth and/or maintenance; regulation of cell growth; signal transduction |
|  |  |  | CHCHD7 | 2.7351 | a nuclear protein expressed in human proliferative cytotrophoblasts and JEG3 choriocarcinoma cells but not in adult tissues |
|  |  |  | URB1 | 2.3679 | is strongly induced and becomes the dominant protein component of old, stationary phase viable cultures |
|  |  |  | C13orf3 | 3.4508 | a novel binding partner of Ska2 by mass spectrometry; the Ska complex has a more central function in mitotic progression than surmised earlier |
|  |  |  | CAPRIN1 | 2.7644 | tightly correlated with cellular proliferation |
|  |  |  | PDS5A | 2.0338 | positive and negative roles in sister chromatid cohesion, possibly by directly modulating the dynamic interaction of cohesin with chromatin |
|  |  |  | MYCT1 | 2.4514 | a novel candidate tumor suppressor; increased proliferation, decreased apoptosis |
|  |  |  | RLN2 | 2.5516 | regulating the growth and remodeling of reproductive tissues in late pregnancy |
|  |  |  | DEPDC1B | 3.2073 | play an essential role in the growth of bladder cancer cells |
|  |  |  | RPL22P15 | 2.0195 | activated a p53-dependent checkpoint that produced a remarkably selective block in αβ T cell development but spared γδ-lineage cells |
|  |  |  | IRX3 | 2.1847 | exploited to drive differentiation of renal progenitors derived from embryonic stem cells toward intermediate tubule fate |
|  |  |  | REG4 | 2.6191 | play a role in tissue regeneration and inflammation in digestive organs; function as trophic or anti-apoptotic factors in cancers |
|  |  |  | CDKN2AIPNL | 2.538 | is CDKN2A interacting protein N-terminal like according to the NCBI gene database, while CDKN2A has been found to encode proteins that inhibit CDK4 kinase |
|  |  |  | MAD2L1 | 2.4011 | essential members of the spindle checkpoint proteins |
|  |  |  | EMP1 | 2.9472 | play important roles in the regulation of cell proliferation, differentiation, and cell death |
|  |  |  | FBXW8 | 2.3389 | plays an essential role in cancer cell proliferation through proteolysis of cyclin D1 |
|  |  |  | BCCIP | 2.6765 | regulate centrosome stability through p53 and p21 functions |
|  |  |  |  |  |  |
|  | **Transcription** |  | KIAA1257 | 2.0954 | activate NR5A1 gene expression; NR5A1 is a transcription factor involved in the development of adrenal/gonadal tissues and steroidogenic linage cell differentiation in adult somatic stem cells |
|  |  |  | MLF1IP | 2.1145 | transcriptional regulator; is an additional factor required for centromere assembly |
|  |  |  | KLF5 | 2.0098 | transcription factor; DNA-binding transcriptional regulator; tumor suppressor; important mediator for the proinflammatory response |
|  |  |  | FMNL3 | 2.2388 | tumor-associated |
|  |  |  | TAF5L | 2.0901 | component of the PCAF histone acetylase complex |
|  |  |  | FOXD1 | 2.0011 | transcription factors; a role in tumor formation |
|  |  |  | MED9 | 2.2934 | coactivator required for activation of RNA polymerase II transcription by DNA bound transcription factors; transcriptional repression |
|  |  |  | FBXO4 | 2.741 | ubiquitin protein ligase complex; tumor suppressor properties; a negative regulator of cyclin D1 |
|  |  |  | EAF2 | 2.1232 | contain transcriptional activation domains; transcriptional elongation factor |
|  |  |  | EIF2AK1 | 2.2561 | translation initiation; a kinase that can be inactivated by hemin |
|  |  |  | UHRF1 | 2.4965 | binds to specific DNA sequences, and recruits a histone deacetylase to regulate gene expression |
|  |  |  | NBR2 | 2.0961 | miscRNA; close proximity on chromosome 17 to tumor suppressor gene BRCA1 |
|  |  |  | ZNHIT2 | 2.1371 | gene regulation and chromatin remodeling |
|  |  |  | KLF6 | 3.1676 | Modulates Recruitment and Polarization of Inflammatory Cells |
|  |  |  | RAD18 | 2.034 | regulate the level of dimethylation of histone H3 at Lys4 and maintain meiotic sex chromosome inactivation; involved in postreplication repair |
|  |  |  | ZCCHC7 | 2.0192 | transcription factors with unknown roles in B lymphoid cells |
|  |  |  | ZSCAN10 | 2.1193 | transcription factor; core transcriptional regulatory network and together with Oct4 and Sox2 regulates differentiation of ESC |
|  |  |  | DLGAP5 | 3.0226 | tumor suppressors |
|  |  |  | DPF3 | 2.5431 | associated with the BAF chromatin remodeling complex and binds methylated and acetylated lysine residues of histone 3 and 4 |
|  |  |  | CBX8 | 2.3923 | a human PcG protein, functioning as a transcription repressor |
|  |  |  | CRY2 | 3.5811 | a potent inhibitor of E-box-dependent gene expression |
|  |  |  | GON4L | 2.5579 | repressed the activity of a nearby promoter; as a platform for the assembly of complexes that regulate gene expression |
|  |  |  | ZNF8 | 2.7411 | as a general inhibitor that blocks the trans-activity of Smad proteins; as a corepressor of Smad1 to actively repress the transcription of BMP downstream genes |
|  |  |  | SLC35B3 | 3.2171 | involved in coordinated transcriptional regulation during acute inflammation |
|  |  |  | ZNF527 | 2.0544 | binding partners of calmodulin |
|  |  |  | INTS7 | 2.3671 | associates with and forms part of the transcriptional regulatory network of human RNA polymerase II |
|  |  |  | NAP1L1 | 2.5956 | a protein that binds acetylated histones and serves as a chaperone to open up chromatin on downstream targets of MyoD/CREBBP |
|  |  |  | RBPMS2 | 2.6206 | involved in regulation of mRNA translation；interact with Smad2, -3, and -4, which regulate TGF-β signaling and with ataxin 1 |
|  |  |  | ZXDA | 2.4483 | an important regulatory complex for MHC II gene transcription |
|  |  |  | SOX4 | 2.124 | as a transcriptional activator, it is capable of binding to the T cell enhencer motif AACAAAg |
|  |  |  | SOX8 | 2.1056 | interaction with the transcription machinery, chromatin remodeling activities or transcriptional co-factors |
|  |  |  | ZNF44 | 2.2787 | play a role in DNA binding |
|  |  |  | ZNF215 | 2.7045 | repress transcription by recruiting corepressors |
|  |  |  | ZNF479 | 2.1388 | recruit histone modifying proteins via interaction with the corepressor KAP1 |
|  |  |  | GTF2A1 | 2.8234 | binds directly to TATA-binding protein and is required for transcriptional activation; interactions of DNA and RNA polymerase |
|  |  |  | NEK9 | 2.566 | a class of chromatin structure modulators with important roles in replication and transcription |
|  |  |  | NR4A3 | 2.3749 | regulate the transcription |
|  |  |  | CENPA | 2.4067 | a histone H3-like protein that is thought to be involved in the nucleosomal packaging of centromeric DNA |
|  |  |  | PCBD2 | 2.7687 | acting on its transcriptional activity; participates in the recycling of tetrahydrobiopterin (BH4) |
|  |  |  | SCML4 | 2.607 | involved in transcriptional regulation |
|  |  |  | NCRNA00115 | 2.0502 | play a role in stress responses |
|  |  |  | TFAM | 2.1447 | maintenance of mitochondrial DNA (mtDNA) |
|  |  |  | PARP4 | 2.6742 | part of vault ribonucleoprotein particles |
|  |  |  | RUVBL1 | 3.7869 | activation of transcription of certain essential genes, and it is involved in recombination repair and/or transcription |
|  |  |  | AUH | 2.6159 | an RNA binding protein with intrinsic enzymatic activity |
|  |  |  | SMC2 | 2.0835 | a group of prokaryotic and eukaryotic chromosomal proteins that are likely to be one of the key components in establishing the ordered structure of chromosomes |
|  |  |  | MACROD2 | 2.5715 | involved in many processes including DNA repair, transcriptional activation and repression, and telomere and chromatin biology |
|  |  |  | UBN2 | 2.1409 | bind directly to both viral and cellular transcription factors |
|  |  |  | COBLL1 | 4.8673 | negative regulators of apoptosis in cultured tumor cells |
|  |  |  | TBX1 | 2.4424 | is required for normal development of the pharyngeal arch arteries in a gene dosage-dependent manner. |
|  |  |  | ELP4 | 2.0095 | involved in transcription and in tRNA modification |
|  |  |  | ZBTB24 | 2.099 | involved in DNA methylation of juxtacentromeric DNA and in B cell development and/or B and T cell interactions |
|  |  |  | HDAC9 | 2.5014 | associate with MEF2 proteins and suppress their transcriptional activity |
|  |  |  | ALS2CR8 | 2.9233 | an emerging regulator of p53 tumor suppressor |
|  |  |  | RANBP6 | 2.3044 | strong candidate oncogenes; are required for the proliferation and survival of lymphoma lines |
|  |  |  |  |  |  |
|  | **Signal transduction** |  | SLC6A5 | 2.9414 | regulating neurotransmitter signaling and homeostasis by mediating uptake of released neurotransmitters from the extracellular space into neurons and glial cells |
|  |  |  | GRIN3A | 2.3463 | are related to glutamate signaling; function in physiological and pathological processes in the central nervous system |
|  |  |  | NDFIP1 | 2.391 | improving neuronal survival during brain injury; participate in a number of cellular trafficking activities including viral budding, protein sorting, and cell signaling |
|  |  |  | CHRNB3 | 2.3287 | signal transmission; are related to smoking behavior and lung cancer |
|  |  |  | RAPGEF2 | 2.4597 | signal transduction as GTP/GDP-regulated switches |
|  |  |  | PIP5K1B | 3.2661 | regulate specific Ca2+ signaling steps; controls RhoA activation |
|  |  |  | DHODH | 2.4336 | modulates transcriptional elongation in the neural crest and melanoma; direct electron acceptor |
|  |  |  | PCDH9 | 3.8492 | a cadherin-related neuronal receptor; specific neuronal connections and signal transduction; involved in specific neuronal connections |
|  |  |  | APBA3 | 2.707 | involved in signal transduction processes |
|  |  |  | RSU1 | 2.1954 | inhibited Jun kinase activation but enhanced Erk2 activation in response to epidermal growth factor; inhibiting the growth of cells |
|  |  |  | HRSP12 | 3.1215 | pathogenetic pathways for MDS patients |
|  |  |  | OTUD4 | 2.65 | related to Dorso-ventral axis formation, Axon guidance, Long-term potentiation, Renal cell carcinoma, TGF-β signaling pathway, T cell receptor signaling pathway, MAPK signaling pathway |
|  |  |  | ANXA2 | 2.8077 | involved in Pathogenesis of Osteoporosis |
|  |  |  | ZDHHC16 | 2.273 | important for protein-protein interactions |
|  |  |  | SYT15 | 2.4811 | involvement in Ca2+-mediated insulin secretion |
|  |  |  | GNB5 | 3.9527 | regulator of G-protein signaling (RGS)-interacting molecule; Rho-signaling cascade |
|  |  |  | 5-Sep | 2.1269 | a target of the neuronal-specific kinase Cdk5 |
|  |  |  | TPX2 | 2.3498 | is phosphorylated during mitosis in a microtubule-dependent way; is nuclear during interphase and becomes localized to spindle poles in mitosis |
|  |  |  | ATP11C | 2.1858 | a multifunctional transporter |
|  |  |  | ROCK2 | 2.24209 | regulating cell movements; attenuates TGF-β signaling by promoting lysosomal degradation of TGF-β type I receptors |
|  |  |  | MAML2 | 2.0203 | modify Notch signaling |
|  |  |  | SLC25A23 | 2.2925 | a mitochondrial subcellular localization when transfected in HeLa cells and is able to bind calcium by Ca2+-dependent mobility shift assays |
|  |  |  | IL2RG | 2.2179 | IL2RG expression allows T-cell development |
|  |  |  | ZFR2 | 2.1949 | RNA binding proteins containing copies of the double-stranded RNA binding motif |
|  |  |  | SHCBP1 | 2.0815 | activated epidermal growth factor receptor |
|  |  |  | MET | 3.0375 | a transmembrane tyrosine kinase receptor; induction of mitogenesis, motogenesis, morphogenesis, metastogenesis and anti-tumor activity on a variety of epithelial cells |
|  |  |  | SEMA4F | 2.3262 | play important roles in axon guidance, in the regulation of cell migration and angiogenesis, and in the modulation of the immune system |
|  |  |  | PBK | 4.9352 | as a Dlg1-interacting protein; involved in mitosis as shown by its significant role in highly proliferating spermatocytes |
|  |  |  | PDSS2 | 2.1397 | as an electron carrier in the mitochondrial respiratory chain, where it transports electrons from complexes I or II to complex III |
|  |  |  | FCRL3 | 3.346 | signaling functions; a pivotal role in autoimmunity |
|  |  |  | ECT2 | 2.8025 | have transforming properties and interact with Rho-like proteins of the Ras superfamily; associates with CYK-4 in a cell cycle–regulated manner |
|  |  |  | NEUROG1 | 2.0266 | as a determinant of neuronal identity and is expressed in a subset of interneuronal precursors of the murine spinal cord in a pattern complementary to Math1 |
|  |  |  | MAGEB2 | 2.2907 | encodes an embryonic antigen normally silenced after birth except in testis and placenta. |
|  |  |  | LRRC34 | 2.2782 | in early mammalian development, neural development, cell polarization, regulation of gene expression and apoptosis signaling |
|  |  |  | ZP3 | 2.1951 | contributes to recognition of spermatozoa during acrosomal exocytosis |
|  |  |  | ATG10 | 4.5633 | a role in autophagy-related processes E2-like enzyme necessary for conjugation of Atg12 to Atg5 |
|  |  |  | ABCG5 | 2.399 | implicated in mediating the secretion of sterols from the liver and efflux of dietary sterols from the gut |
|  |  |  | DARS2 | 4.0783 | related to metastasis potential of murine hepatocarcinoma; Leukoencephalopathy with brain stem and spinal cord involvement |
|  |  |  | PSORS1C1 | 2.1144 | Systemic sclerosis (SSc) is a connective tissue disease characterized by generalized microangiopathy, severe immunologic alterations, and massive deposits of matrix components in the connective tissue; a large genome-wide association study of SSc and identified two new SSc-risk loci, PSORS1C1 and TNIP1 |
|  |  |  | SRGAP2 | 2.4068 | negatively regulates neuronal migration and induces neurite outgrowth |
|  |  |  | OR6M1 | 2.3433 | G protein-coupled transmembrane receptors; involved in sperm maturation, migration or fertilization |
|  |  |  | TTK | 3.3213 | identified by screening of a T cell expression library with anti-phosphotyrosine antibodies |
|  |  |  | SHOX | 2.7054 | The homeobox gene, SHOX, in the pseudoautosomal region is the major player and that haploinsuffiency of this gene leads to the growth failure seen in Turner syndrome |
|  |  |  | BCS1L | 2.1258 | a member of the AAA family of ATPases that is necessary for the assembly of complex III in the mitochondria |
|  |  |  | MSLNL | 2.339 | associated with the evolution of the olfactory system in African great apes including human |
|  |  |  | TBC1D16 | 3.2607 | a Rab GTPase-activating protein of unknown function |
|  |  |  | CCDC85C | 3.0077 | an important role in cortical development, especially in the maintenance of radial glia |
|  |  |  | SPAG9 | 2.274 | involved in transducing signals during the gamete fusion |
|  |  |  | PMCH | 2.0552 | MCH plays an inhibitory role in regulating locomotor activity |
|  |  |  | CHEK1 | 2.5369 | Activated CHEK1 can phosphorylate and modulate the activity a number of proteins including p53, providing a link between ATR sensing of DNA damage and p53 checkpoint activity. |
|  |  |  | OR6K2 | 2.5041 | G protein-coupled seven-transmembrane proteins; induces a cascade of intracellular events resulting in an influx of both Na+ and Ca2+ that culminate in the generation of a graded receptor potential in the soma of the sensory neuron |
|  |  |  | GPR113 | 2.1238 | as a receptor for an ingested peptide(s), many of which are known to elicit taste sensations |
|  |  |  | CYP4F2 | 2.3326 | important role in regulating circulating as well as hepatic levels of this powerful proinflammatory eicosanoid |
|  |  |  | PGDS | 2.1809 | play important physiological roles in the placenta; regulatory role in the processes of parturition |
|  |  |  | S100A5 | 2.5058 | a high affinity for Ca2+, Zn2+, and Cu2+ and that it induces distinct structural changes |
|  |  |  | AGBL2 | 2.8353 | ATP/GTP binding protein-like 2 (AGBL2; a carboxypeptidase) and RARRES1 (an inhibitor) regulate the tubulin tyrosination cycle |
|  |  |  | CATSPER3 | 2.2917 | channel-like proteins; involved in acrosome reaction |
|  |  |  |  |  |  |
|  | **Cytoskeleton** |  | BFSP2 | 2.2229 | cytoskeletal proteins |
|  |  |  | ABI2 | 2.1423 | Rac-dependent cytoskeletal reorganization; participate in Abl kinase signaling in the nervous system |
|  |  |  | PLS3 | 2.0259 | cytoskeleton proteins; an actin-bundling protein |
|  |  |  | CENPM | 4.1275 | an additional factor required for centromere assembly |
|  |  |  | NDC80 | 2.6638 | a component of the NDC80 kinetochore complex; organize and stabilize microtubule-kinetochore interactions and is required for proper chromosome segregation |
|  |  |  | MAP9 | 3.0525 | a centrosome- and spindle-associated protein; microtubule-associated protein required for spindle function, mitotic progression, and cytokinesis |
|  |  |  | CENPK | 4.5873 | centromeric complex; kinetochore function and mitotic progression |
|  |  |  | KIF20B | 2.3112 | M phase phosphoprotein 1; mitotic molecular motor required for completion of cytokinesis；specifically phosphorylated at the G2/M transition |
|  |  |  | KIF1B | 2.6115 | microtubule-dependent molecular motors involved in the intracellular motile process; the transport of synaptic vesicles in neuronal cells |
|  |  |  | KIF2C | 2.1757 | the best-characterized member of the family and localizes to spindle poles, spindle midzone, and kinetochores in addition to associating with the tips of growing microtubules |
|  |  |  | KIF18B | 2.7901 | a cell cycle-dependent manner and therefore may play an important role(s) in cell division |
|  |  |  | FRMD4A | 2.3747 | a scaffolding protein; a central player in actin cytoskeleton dynamics and membrane trafficking, during junctional remodeling and epithelial polarization |
|  |  |  | ICA1 | 2.1569 | cellular trafficking; an autoantigen in insulin-dependent diabetes mellitus and primary Sjogren's syndrome |
|  |  |  | CKAP2 | 2.2384 | microtubule-associated protein |
|  |  |  | WDR51A | 2.8077 | recruited to the centriole |
|  |  |  | LIMA1 | 2.4071 | actin binding |
|  |  |  | MAP7 | 2.1225 | play a role in mature rather than immature epithelial cells and may contribute to epithelial polarity; is associated with cytoskeletal filaments |
|  |  |  | WWC1 | 2.7333 | a linker molecule between polarity proteins and components of the cytoskeleton |
|  |  |  | ANKRD44 | 2.6088 | plays an important role during mitotic spindle formation |
|  |  |  | ACTN3 | 2.5779 | be important for anchoring actin and playing a regulatory function in coordinating muscle fiber contraction |
|  |  |  | CCDC52 | 2.1509 | is required for centriole duplication, and for proper bipolar spindle formation and chromosome congression in mitosis |
|  |  |  | CNO | 2.1247 | play a role in cytoskeletal organization and/or establishment of cell polarity |
|  |  |  | tcag7.1239 | 2.4948 | cytoplasmic protein; plays a functional role in the cell filament networks or the transport of vesicles |
|  |  |  | ZWINT | 3.3111 | a critical role of Zwint-1 in kinetochore assembly dynamics |
|  |  |  | C18orf24 | 2.8783 | prolonged mitotic delay even though most chromosomes are aligned in a near-perfect metaphase plate |
|  |  |  | SGOL2 | 2.157 | protecting centromeric cohesion; SGOL2 is essential for meiosis |
|  |  |  | KRT2 | 2.9545 | an important role in the mechanical support of hair development |
|  |  |  | C8orf37 | 2.0288 | Encoding a Ciliary Protein, are Associated with Autosomal-Recessive Retinal Dystrophies with Early Macular Involvement |
|  |  |  | EPB49 | 3.503 | the actin filaments may depolymerize up to the membrane |
|  |  |  | TSGA10 | 2.1081 | plays a role in the sperm tail fibrous sheath |
|  |  |  | MYO3A | 3.1064 | plays a structural role in the CPs(microvillus-like calycal processes) |
|  |  |  | ABLIM2 | 2.3809 | as STARS-interacting proteins; as a scaffold for signaling modules of the actin cytoskeleton and thereby modulate transcription |
|  |  |  | RBL1 | 2.0921 | regulate telomere length |
|  |  |  | TP63 | 2.8834 | required for maintenance of diverse epithelial and apocrine structures |
|  |  |  | VMAC | 2.9347 | cytoskeletons of mesenchymal cells of vertebrates |
|  |  |  | MYO1E | 2.0996 | colocalizes with actin in actively extending regions of the cell, crown-like structures and the phagocytic apparatus |
|  |  |  | NUP93 | 2.1552 | participate in the establishment of the NPC resting pore size |
|  |  |  | MAP4 | 2.232 | be important in targeting the mitotic kinase to appropriate cytoskeletal substrates, for the regulation of spindle assembly and dynamics. |
|  |  |  | TMPO | 2.5281 | the regulation of nuclear architecture by binding lamin B1 and chromosomes in a manner regulated by phosphorylation during mitosis |
|  |  |  | KRTAP11-1 | 2.4236 | Intermediate filament (IF) keratins and keratin-associated proteins (KAPs) are principal structural components of hair and encoded by members of multiple gene families |
|  |  |  |  |  |  |
|  | **Biosynthesis of macromolecules** |  | ISCA1 | 2.1481 | biogenesis and assembly of iron-sulfur clusters |
|  |  |  | COX11 | 2.9356 | assembly of an active cytochrome c oxidase complex |
|  |  |  | RLBP1 | 2.5586 | a functional component of the visual cycle |
|  |  |  | TMEM182 | 3.2698 | adipogenesis and myogenesis |
|  |  |  | KIAA1609 | 2.1 | influence glucose metabolism in the context of insulin resistance |
|  |  |  | QKI | 2.2301 | regulate RNA metabolism of several myelin structural genes as well as differentiation of progenitor cells into oligodendrocytes |
|  |  |  | HAS3 | 2.2679 | involved in the synthesis of the unbranched glycosaminoglycan hyaluronan, or hyaluronic acid; regulator of hyaluronan synthesis |
|  |  |  | MRPL52 | 2.1529 | protein synthesis within the mitochondrion |
|  |  |  | ETNK1 | 2.0038 | primarily important for the biosynthesis of phosphatidylethanolamine |
|  |  |  | SPR | 2.3663 | involved in the biosynthesis of tetrahydrobiopterin |
|  |  |  | LARS | 3.2855 | is essential to the fidelity of protein synthesis and hence cell survival |
|  |  |  |  |  |  |
| **Down-regulated** | |  |  |  |  |
|  | **Immune response** | -ILs | IL1A | 0.0519 | is responsible for the production of inflammation; induces apoptosis |
|  |  |  | IL1B | 0.0424 | an important mediator of the inflammatory response; involved in cell proliferation, differentiation, and apoptosis |
|  |  |  | IL2RA | 0.4324 | Receptor for interleukin-2 |
|  |  |  | IL8 | 0.0428 | one of the major mediators of the inflammatory response |
|  |  |  | IL17C | 0.358 | stimulates the release of tumor necrosis factor alpha and IL-1-beta from the monocytic cell line THP-1 |
|  |  |  | IL26 | 0.4495 | induces rapid phosphorylation of the transcription factors STAT1 and STAT3, which enhance IL-10 and IL-8 secretion and as expression of the CD54 molecule on the surface of epithelial cells |
|  |  | -TNFs | TNFAIP2 | 0.3504 | a role as a mediator of inflammation and angiogenesis |
|  |  |  | TNFAIP6 | 0.1657 | involved in cell-cell and cell-matrix interactions during inflammation and tumorigenesis |
|  |  |  | TNFSF14 | 0.2955 | Activates NFKB, stimulates the proliferation of T-cells, and inhibits growth of the adenocarcinoma HT-29 |
|  |  | -IFNs | IFNA10 | 0.3704 | produced by macrophages, IFN-alpha have antiviral activities |
|  |  | -Chemokines | CCL3 | 0.0516 | plays a role in inflammatory responses |
|  |  |  | CCL4 | 0.1015 | Monokine with inflammatory and chemokinetic properties; plays a central role in the normal initiation of T cell and humoral responses |
|  |  |  | CCL20 | 0.0185 | important roles in the initiation of immune responses; expressed on dendritic cells , memory T cells , and NK cells |
|  |  |  | CCL23 | 0.1129 | involved in immunoregulatory and inflammatory processes |
|  |  |  | CCL26 | 0.4822 | involved in immunoregulatory and inflammatory processes |
|  |  |  | CXCL1 | 0.0598 | roles in the development, homeostasis, and function of the immune system; play a role in inflammation and exerts its effects on endothelial cells in an autocrine fashion |
|  |  |  | CXCL3 | 0.1118 | play a role in inflammation and exert its effects on endothelial cells in an autocrine fashion |
|  |  |  | CXCR7 | 0.2424 | promotes B-cell retention in the splenic marginal zone and serves as a sink for CXCL12 |
|  |  | -Others | CD86 | 0.2981 | involved in the costimulatory signal essential for T-lymphocyte proliferation and interleukin-2 production, by binding CD28 or CTLA-4 |
|  |  |  | NFKBIZ | 0.3167 | a role in inflammatory responses to LPS |
|  |  |  | RNF19B | 0.4294 | involved in the cytolytic function of NK cells and CTLs |
|  |  |  | SEMA7A | 0.2573 | an extremely potent monocyte activator; stimulating chemotaxis and inflammatory cytokine production |
|  |  |  | C1S | 0.4983 | encodes a serine protease, which is a major constituent of the human complement subcomponent C1 |
|  |  |  | PML | 0.4825 | play a key role also in immune response to viral infection; the innate immune Toll-like receptor (TLR)/NF-κB prosurvival pathway |
|  |  |  | ICAM1 | 0.3217 | a cell surface glycoprotein which is typically expressed on endothelial cells and cells of the immune system |
|  |  |  | ZFP36 | 0.4523 | a component of a negative feedback loop that interferes with TNF-alpha production by destabilizing its mRNA |
|  |  |  | FREQ | 0.1126 | calcium-binding proteins expressed predominantly in neurons |
|  |  |  | CD80 | 0.3978 | The costimulatory signal essential for T-lymphocyte activation |
|  |  |  | ZNF175 | 0.3885 | Interferes with HIV-1 replication by suppressing Tat-induced viral LTR promoter activity |
|  |  |  | DEFA3 | 0.4641 | have antibiotic, fungicide and antiviral activities |
|  |  |  | NRP1 | 0.3381 | a specific surface marker for CD4+CD25+ Tr cells; a receptor involved in axon guidance, angiogenesis, and the activation of T cells |
|  |  |  | OSM | 0.1146 | regulates cytokine production |
|  |  |  | GNL1 | 0.4357 | possible regulatory or functional link with the histocompatibility cluster |
|  |  |  | CD300LB | 0.4994 | acts as an activating immune receptor through its interaction with ITAM-bearing adapter TYROBP, and also independently by recruitement of GRB2 |
|  |  |  | NOS1 | 0.3463 | involved in immunity and inflammation |
|  |  |  | LGMN | 0.3893 | involved in the processing of bacterial antigens for MHC class II presentation |
|  |  |  |  |  |  |
|  | **Transportation and metabolism** |  |  |  |  |
|  |  | - Energy metabolism and oxidation | TRPV4 | 0.3595 | involved in the regulation of systemic osmotic pressure |
|  |  |  | AQP9 | 0.3905 | stimulates urea transport, osmotic water permeability and glycerol permeability; play a role in specialized leukocyte functions |
|  |  | -Lipid metabolism | APOL3 | 0.491 | affect the movement of lipids in the cytoplasm or allow the binding of lipids to organelles |
|  |  |  | FFAR2 | 0.2724 | a receptor; involved in the inflammatory response and in regulating lipid plasma levels |
|  |  | - Signal transduction | TMEM35 | 0.4476 | interact with NGFR and modulate neurite outgrowth |
|  |  |  | ABCB1 | 0.4707 | as a transporter in the blood-brain barrier; regulation of pro-inflammatory cytokine secretion |
|  |  |  | OLR1 | 0.2361 | play a role as a scavenger receptor |
|  |  |  | CLCA3P | 0.4306 | this gene is unlikely to be protein-coding |
|  |  |  | TOM1L2 | 0.3294 | Probable role in protein transport; May regulate growth factor-induced mitogenic signaling |
|  |  |  | BEST3 | 0.1988 | belongs to the bestrophin family of anion channels |
|  |  |  | P2RY6 | 0.2219 | inhibits effector T cell activation in allergic pulmonary inflammation |
|  |  |  | CDH7 | 0.4692 | calcium dependent cell adhesion proteins |
|  |  | - Protein transportation and metabolism | SNAI1 | 0.1696 | SNAI1 accelerates metastasis not only by enhancing invasion but also by inducing multiple immunosuppression mechanisms |
|  |  |  | SLCO4A1 | 0.2107 | solute carrier organic anion transporter |
|  |  |  | NCAM1 | 0.4602 | involved in the expansion of T cells and dendritic cells which play an important role in immune surveillance |
|  |  |  | SEMA3F | 0.4256 | play a role in cell motility and cell adhesion; involved in immune responses, cancer progression, metastasis and angiogenesis |
|  |  |  | HSPA5 | 0.4128 | play a key role in monitoring protein transport through the cell |
|  |  |  | CABP7 | 0.1892 | negatively regulates Golgi-to-plasma membrane trafficking by interacting with PI4KB and inhibiting its activity; regulate key ion channels in the mammalian nervous system |
|  |  |  | RAB9B | 0.4662 | involved in endosome-to-Golgi transport |
|  |  |  | VPS18 | 0.3312 | play a role in vesicle-mediated protein trafficking to lysosomal compartments |
|  |  |  | ABCG1 | 0.4555 | transporters; ABCG1 deletion in macrophages causes a striking inflammatory phenotype and initiates onset of pulmonary lipidosis |
|  |  |  | RIMS3 | 0.4244 | Regulates synaptic membrane exocytosis |
|  |  |  | NFE2L1 | 0.2802 | Activates erythroid-specific, globin gene expression |
|  |  |  | ABI3 | 0.466 | inhibits ectopic metastasis of tumor cells as well as cell migration |
|  |  |  | NUP98 | 0.497 | play a role in the bidirectional transport across the nucleoporin complex (NPC) |
|  |  |  | AP1S3 | 0.461 | a role in protein sorting in the late-Golgi/trans-Golgi network (TGN) and/or endosomes |
|  |  |  | GPR97 | 0.4874 | Orphan receptor |
|  |  |  | MERTK | 0.2808 | In case of filovirus infection, seems to function as a cell entry factor |
|  |  |  | TM7SF4 | 0.1901 | a transmembrane molecule that is preferentially expressed by dendritic cells |
|  |  |  | SNX8 | 0.4497 | involved in several stages of intracellular trafficking; play a role in intracellular protein transport from early endosomes to the trans-Golgi network |
|  |  |  | PPP1R10 | 0.4481 | scaffold protein; Inhibitor of PPP1CA and PPP1CC phosphatase activities |
|  |  |  | SLC1A4 | 0.4392 | as receptors |
|  |  |  | SLC12A7 | 0.4916 | potassium: chloride symporter activity |
|  |  |  | SLC16A3 | 0.4467 | Proton-linked monocarboxylate transporter; catalyzes the rapid transport across the plasma membrane of many monocarboxylates |
|  |  |  | SLC22A4 | 0.4549 | Sodium-ion dependent, low affinity carnitine transporter |
|  |  |  | SLC26A11 | 0.4814 | a novel Na+-independent sulfate transporter that may cooperate with SLC26A2 to mediate DIDS-sensitive sulfate uptake into HEVEC |
|  |  |  | SLC41A2 | 0.3578 | acts as a plasma-membrane magnesium transporter |
|  |  |  | SLC43A2 | 0.4892 | mediate sodium-independent transport of bulky neutral amino acids across cell membranes |
|  |  |  | SLC46A2 | 0.3372 | May act as a transporter |
|  |  |  | LFNG | 0.4632 | a single-pass type II Golgi membrane protein |
|  |  |  |  |  |  |
|  | **Cell proliferation** | - DNA replication | NEIL2 | 0.4204 | initiate the first step in base excision repair by cleaving bases damaged by reactive oxygen species and introducing a DNA strand break via the associated lyase reaction |
|  |  |  | PPP2R5C | 0.4236 | play a role in DNA damage-induced inhibition of cell proliferation |
|  |  | - Translation | BASP1 | 0.4705 | a novel target suppressed by Myc |
|  |  |  | CABLES1 | 0.4457 | Plays a role as a regulator for p53/p73-induced cell death |
|  |  | - cell cycle | NEK6 | 0.4028 | is required for cell cycle progression |
|  |  |  | NUPR1 | 0.3119 | implicated in the regulation of cell cycle and apoptosis |
|  |  |  | G0S2 | 0.3006 | is required to commit cells to enter the G1 phase of the cell cycle |
|  |  |  | PELO | 0.4643 | a role in spermatogenesis, cell cycle control, and in meiotic cell division |
|  |  |  | SMPD3 | 0.4619 | regulates the cell cycle by acting as a growth suppressor in confluent cells |
|  |  |  | ZAK | 0.2765 | plays a role in cell cycle checkpoint regulation in cells |
|  |  | - Ubiquitin-proteosome | ACHE | 0.4992 | role in neuronal apoptosis |
|  |  |  | RNF103 | 0.4668 | a ubiquitin ligase |
|  |  |  | RNF122 | 0.4829 | induce necrosis and apoptosis; play a role in cell viability |
|  |  |  | NR4A1 | 0.3482 | induces apoptosis; inhibit NF-kappa-B transactivation of IL2 |
|  |  |  | DIDO1 | 0.3506 | involved in apoptosis |
|  |  |  | ARHGEF7 | 0.4934 | as a positive regulator of apoptosis; function in cell migration |
|  |  |  | TRPC4AP | 0.4094 | Substrate-specific adapter of a DCX (DDB1-CUL4-X-box) E3 ubiquitin-protein ligase complex required for cell cycle control |
|  |  |  | SH3RF2 | 0.2969 | May be a E3 ubiquitin-protein ligase (Potential) |
|  |  |  | SMURF2 | 0.4589 | accepts ubiquitin from an E2 ubiquitin-conjugating enzyme |
|  |  |  | UNKL | 0.4948 | participate in a protein complex showing an E3 ligase activity regulated by RAC1 |
|  |  | - Signal transduction | TUT1 | 0.4399 | a nucleotidyl transferase; controlling gene expression and cell proliferation |
|  |  |  | SLC7A5 | 0.4911 | a role in neuronal cell proliferation (neurogenesis) in brain |
|  |  |  | SRC | 0.4259 | play a role in the regulation of embryonic development and cell growth |
|  |  |  | PSEN1 | 0.4769 | involved in the development of the brain and spinal cord (central nervous system) and the survival of nerve cells (neurons) |
|  |  |  | ERCC4 | 0.4167 | nucleotide Excision Repair (NER) proteins |
|  |  |  | GADD45G | 0.2482 | Involved in the regulation of growth and apoptosis |
|  |  |  | FGF18 | 0.46 | Stimulates hepatic and intestinal proliferation |
|  |  |  | SDC2 | 0.2176 | as an integral membrane protein and participates in cell proliferation, cell migration and cell-matrix interactions via its receptor for extracellular matrix proteins |
|  |  |  | DAPK1 | 0.483 | a positive mediator of gamma-interferon induced programmed cell death |
|  |  |  | HBEGF | 0.3994 | involved in macrophage-mediated cellular proliferation; acts as a diphtheria toxin receptor |
|  |  |  | PID1 | 0.3503 | Increases proliferation of preadipocytes without affecting adipocytic differentiation |
|  |  |  | PARD6G | 0.2451 | involved in asymmetrical cell division and cell polarization processes; play a role in the formation of epithelial tight junctions |
|  |  |  | S1PR2 | 0.4923 | participates in sphingosine 1-phosphate-induced cell proliferation, survival, and transcriptional activation |
|  |  |  | AHI1 | 0.384 | required for both cerebellar and cortical development in humans |
|  |  |  | IER3 | 0.1481 | play a role in the ERK signaling pathway by inhibiting the dephosphorylation of ERK by phosphatase PP2A-PPP2R5C holoenzyme |
|  |  |  | CLN8 | 0.3883 | function in lipid synthesis, transport, or sensing; play a role in cell proliferation during neuronal differentiation and in protection against cell death |
|  |  |  | RERG | 0.3853 | inhibits cell proliferation and tumor formation |
|  |  |  | NOTCH4 | 0.4513 | as a receptor; regulate branching morphogenesis in the developing vascular system |
|  |  |  | USP11 | 0.4562 | controls many intracellular processes, including cell cycle progression, transcriptional activation, and signal transduction |
|  |  |  |  |  |  |
|  | **Transcription** |  | NFKB2 | 0.4119 | regulate peripheral lymphoid organogenesis and B-lymphocyte differentiation |
|  |  |  | FBXO28 | 0.4541 | an important regulator of Myc-driven transcription through the ubiquitin-dependent recruitment of a transcriptional cofactor to Myc target gene promoters |
|  |  |  | ING5 | 0.4801 | a tumor suppressor protein; involvement in TP53-dependent regulatory pathway |
|  |  |  | CHD6 | 0.4271 | as a negative modulator of influenza virus replication |
|  |  |  | ZNF732 | 0.4833 | involved in transcriptional regulation |
|  |  |  | VDR | 0.4275 | involved in the immune response and cancer |
|  |  |  | KCNQ1OT1 | 0.4911 | plays an important role in the transcriptional silencing of the KCNQ1 locus by regulating histone methylation |
|  |  |  | LHX5 | 0.4282 | as a transcriptional regulator and be involved in the control of differentiation and development of the forebrain |
|  |  |  | BCOR | 0.3862 | specifically inhibit gene expression when recruited to promoter regions by sequence specific DNA-binding proteins |
|  |  |  | RGS12 | 0.3381 | as a guanosine triphosphatase (GTPase)-activating protein as well as a transcriptional repressor; Inhibits signal transduction |
|  |  |  | KIAA1967 | 0.4815 | inhibits SIRT1 deacetylase activity leading to increasing levels of p53/TP53 acetylation and p53-mediated apoptosis |
|  |  |  | SOX5 | 0.4214 | transcription factors involved in the regulation of embryonic development and in the determination of the cell fate |
|  |  |  | MYO1C | 0.3488 | involved in regulation of transcription; transcription initiation |
|  |  |  | PAX6 | 0.3426 | Transcription factor with important functions in the development of the eye, nose, central nervous system and pancreas |
|  |  |  | RASD1 | 0.3258 | play a role in regulating B lymphocyte activity and proliferation |
|  |  |  | ZNF224 | 0.485 | involved in transcriptional regulation as a transcriptional repressor |
|  |  |  | RYBP | 0.4771 | inhibits ubiquitination and subsequent degradation of TP53, and thereby plays a role in regulating transcription of TP53 target genes |
|  |  |  | EGR4 | 0.4051 | transcriptional regulator |
|  |  |  | ZNF445 | 0.4651 | involved in transcriptional regulation |
|  |  |  | ZHX2 | 0.4171 | acts as a transcriptional repressor |
|  |  |  | HES6 | 0.4878 | a cofactor, interacting with other transcription factors |
|  |  |  | NFKBIA | 0.413 | stimulation by immune and proinflammatory responses |
|  |  |  | IKZF4 | 0.4604 | transcriptional repressor |
|  |  |  | FOXD2 | 0.4539 | transcription factor involved in embryogenesis and somatogenesis |
|  |  |  | ZNF165 | 0.4564 | involved in transcriptional regulation |
|  |  |  | ZNF469 | 0.481 | as a transcription factor or extra-nuclear regulator factor for the synthesis or organization of collagen fibers |
|  |  |  | XAB2 | 0.4771 | a multifunctional factor involved in pre-mRNA splicing, transcription, and transcription-coupled DNA repair |
|  |  |  | IRAK2 | 0.4507 | essential for Toll-like receptor-mediated transcriptional and post-transcriptional regulation of tumor necrosis factor alpha |
|  |  |  | FOXD4 | 0.4298 | reduced DNA binding capacity and altered transcriptional activity |
|  |  |  | BRD1 | 0.4926 | Component of the MOZ/MORF complex which has a histone H3 acetyltransferase activity |
|  |  |  | ZNF835 | 0.4469 | involved in transcriptional regulation |
|  |  |  | PIP5K1A | 0.4065 | regulating mRNA polyadenylation of a select set of mRNAs |
|  |  |  | ZNF613 | 0.4023 | involved in transcriptional regulation |
|  |  |  | KCTD1 | 0.285 | repress the transcriptional activity of AP-2 family members, including TFAP2A, TFAP2B and TFAP2C to various extent |
|  |  |  | ZC3H12A | 0.4047 | Modulates the immune response and inflammation by regulating the decay of specific mRNA molecules |
|  |  |  | ZNF629 | 0.4958 | involved in transcriptional regulation |
|  |  |  | HMBOX1 | 0.1929 | transcription factor |
|  |  |  | ZNF251 | 0.4895 | involved in transcriptional regulation |
|  |  |  |  |  |  |
|  | **Signal transduction** |  | EDN1 | 0.404 | a key mediator in vascular tone and renal homeostatsis |
|  |  |  | PPP1R15A | 0.4775 | plays a critical role in antiviral defense |
|  |  |  | LRP6 | 0.4195 | regulate effector T-cell development, regulatory T-cell activation and dendritic-cell maturation |
|  |  |  | MAPK8 | 0.4514 | a key role in T cell proliferation, apoptosis and differentiation |
|  |  |  | GPR75 | 0.3446 | cell surface receptors that activate guanine-nucleotide binding proteins upon the binding of a ligand |
|  |  |  | NTSR1 | 0.3816 | belongs to the large superfamily of G-protein coupled receptors |
|  |  |  | SLC9A8 | 0.4566 | Plays an important role in signal transduction |
|  |  |  | RASA3 | 0.401 | stimulates the GTPase activity |
|  |  |  | RRAD | 0.4013 | Regulates voltage-dependent L-type calcium channel subunit alpha-1C trafficking to the cell membrane |
|  |  |  | PHACTR1 | 0.4577 | a key regulator of endothelial cell function properties |
|  |  |  | LIF | 0.432 | a role in immune tolerance at the maternal-fetal interface |
|  |  |  | PTPRS | 0.3773 | be signaling molecules that regulate a variety of cellular processes including cell growth, differentiation, mitotic cycle, and oncogenic transformation |
|  |  |  | SPHK1 | 0.4474 | a lipid mediator with both intra- and extracellular functions |
|  |  |  | GPR34 | 0.4401 | mediate signals to the interior of the cell via activation of heterotrimeric G proteins that in turn activate various effector proteins, ultimately resulting in a physiologic response |
|  |  |  | PIK3R5 | 0.4362 | recruits and activates cytosolic effectors involved in proliferation, survival, or chemotaxis. PIK3R5 is a PI3K regulatory subunit |
|  |  |  | CYP1B1 | 0.3462 | participant in eye development |
|  |  |  | LPCAT1 | 0.393 | playing a pivotal role in respiratory physiology |
|  |  |  | CSNK1D | 0.3965 | Participates in Wnt signaling |
|  |  |  | NKD2 | 0.323 | activate a second Wnt signaling pathway |
|  |  |  | DKKL1 | 0.4034 | The dickkopf protein family interacts with the Wnt signaling pathway |
|  |  |  | WNT1 | 0.3474 | is also a ligand for the coreceptor RYK, thus triggering Wnt signaling |
|  |  |  | DKK3 | 0.3699 | inhibit Wnt regulated processes |
|  |  |  | DKK2 | 0.2138 | act as either an agonist or antagonist of Wnt/beta-catenin signaling |
|  |  |  | TMEM88 | 0.4427 | associates with Dvl proteins and regulates Wnt signaling in a context-dependent manner |
|  |  |  | SPSB1 | 0.2155 | the only SPSB family member to be regulated by the same TLR pathways that induce iNOS expression and characterize the interaction between SPSB1 and iNOS |
|  |  |  | ANO9 | 0.4612 | act as a calcium-activated chloride channel |
|  |  |  | AATK | 0.2847 | essential for neuronal differentiation |
|  |  |  | OVGP1 | 0.3657 | play a role in the fertilization process and/or early embryonic development |
|  |  |  | RAMP1 | 0.2124 | acts as a receptor for calcitonin-gene-related peptide (CGRP) together with CALCRL |
|  |  |  | NLRP3 | 0.4758 | as an upstream activator of NF-kappa-B signaling\ Activates caspase-1 |
|  |  |  | SCN4B | 0.4067 | interact with voltage-gated alpha subunits to change sodium channel kinetics |
|  |  |  | PDE4DIP | 0.3086 | as an anchor sequestering components of the cAMP-dependent pathway to Golgi and/or centrosomes |
|  |  |  | GABRQ | 0.3372 | a multisubunit chloride channel that mediates the fastest inhibitory synaptic transmission in the central nervous system |
|  |  |  | LOC553158 | 0.4354 | contains domains characteristic of a RhoGAP protein |
|  |  |  | ROR1 | 0.301 | a receptor protein tyrosine kinase that modulates neurite growth in the central nervous system |
|  |  |  | CNR1 | 0.2399 | involved in cannabinoid-induced CNS effects |
|  |  |  | SAPS1 | 0.2638 | involved in the PP6-mediated dephosphorylation of NFKBIE opposing its degradation in response to TNF-alpha |
|  |  |  | RND1 | 0.3176 | regulate the organization of the actin cytoskeleton in response to extracellular growth factors |
|  |  |  | ADAMTS10 | 0.4327 | important roles in connective tissue organization, coagulation, inflammation, arthritis, angiogenesis and cell migration |
|  |  |  | DLX1 | 0.4357 | play a regulatory role in the development of the ventral forebrain |
|  |  |  | GRIPAP1 | 0.4961 | a guanine nucleotide exchange factor for the Ras family of small G proteins (RasGEF) |
|  |  |  | GEM | 0.4118 | participating in receptor-mediated signal transduction |
|  |  |  | PHLDA2 | 0.4873 | Plays a role in regulating placenta growth |
|  |  |  | ITGA5 | 0.2935 | a receptor for fibronectin and fibrinogen |
|  |  |  | SH2D1B | 0.4218 | controlling signal transduction |
|  |  |  | VCX3A | 0.3516 | play a role in sex ratio distortion |
|  |  |  | RASAL1 | 0.4604 | inhibitory regulator of the Ras-cyclic AMP pathway |
|  |  |  | IGFBP5 | 0.4585 | inhibit or stimulate the growth promoting effects of the IGFs on cell culture |
|  |  |  | CYP1A1 | 0.3322 | involved in an NADPH-dependent electron transport pathway |
|  |  |  | STAT5A | 0.4537 | a member of the STAT family of transcription factors |
|  |  |  | VGLL2 | 0.3458 | act as a specific coactivator for the mammalian TEFs; play a role in the development of skeletal muscles |
|  |  |  | ABHD12 | 0.4715 | involved in a wide range of physiological processes, including neurotransmission, mood, appetite, pain appreciation, addiction behavior, and inflammation |
|  |  |  | OR2A20P | 0.3856 | a pseudogene. |
|  |  |  | LRG1 | 0.3311 | involved in protein-protein interaction, signal transduction, and cell adhesion and development |
|  |  |  | SEMA6B | 0.2311 | play a major role in axon guidance; involved in both peripheral and central nervous system development |
|  |  |  | PRG2 | 0.4244 | involved in antiparasitic defense mechanisms and immune hypersensitivity reactions |
|  |  |  | BMF | 0.4743 | as an apoptotic activator |
|  |  |  | SH2D2A | 0.4771 | an adaptor protein thought to function in T-cell signal transduction |
|  |  |  | SCT | 0.4719 | regulate the growth and development of the stomach, small intestine, and pancreas |
|  |  |  | NINJ1 | 0.4454 | is upregulated after nerve injury both in dorsal root ganglion neurons |
|  |  |  | INPP5A | 0.4664 | acts as a second messengermediating cell responses to various stimulation |
|  |  |  | ENG | 0.3152 | component of the transforming growth factor beta receptor complex |
|  |  |  | RAP1GAP | 0.4129 | a GTPase-activating-protein (GAP); plays a role in diverse processes such as cell proliferation, adhesion, differentiation, and embryogenesis |
|  |  |  | YPEL4 | 0.253 | a nuclear protein; inhibit YPEL4's ability to activate Elk-1 in the MAPK signaling pathway |
|  |  |  | PIP5KL1 | 0.0888 | Acts as a scaffold for localization and activation of PIPKs |
|  |  |  | KLKB1 | 0.4219 | the trypsin family of serine proteases |
|  |  |  | RSPH4A | 0.4219 | a signal transduction scaffold between the central pair of microtubules and dynein |
|  |  |  | LY6G5B | 0.4407 | involved in signal transduction |
|  |  |  | PIK3IP1 | 0.4714 | Negative Regulator of PI3K |
|  |  |  | SCML1 | 0.4497 | involved in spermatogenesis during sexual maturation |
|  |  |  | POM121L9P | 0.3659 | rapid turnover by the nonsense-mediated decay pathway |
|  |  |  | VCX | 0.397 | mediate a process in spermatogenesis; play a role in sex ratio distortion |
|  |  |  | GRAP | 0.4123 | couples signals from receptor and cytoplasmic tyrosine kinases to the Ras signaling pathway |
|  |  |  | LAYN | 0.4102 | receptor for hyaluronate |
|  |  |  | LYPD3 | 0.3634 | supports cell migration; involved in urothelial cell-matrix interactions |
|  |  |  | GRIN1 | 0.4142 | plays a key role in synaptic plasticity, synaptogenesis, excitotoxicity, memory acquisition and learning |
|  |  |  | PCDHB9 | 0.4892 | a critical role in the establishment and function of specific cell-cell neural connections |
|  |  |  | EPHA4 | 0.4972 | play a role in a signal transduction process involved in hindbrain pattern formation |
|  |  |  | EPB41L3 | 0.2618 | critical growth regulator in the pathogenesis of meningiomas |
|  |  |  | SLA2 | 0.4281 | role in downregulating T and B cell-mediated responses and inhibits antigen receptor-induced calcium mobilization |
|  |  |  | TLR8 | 0.435 | leading to NF-kappa-B activation, cytokine secretion and the inflammatory response |
|  |  |  | TNNT3 | 0.3137 | required for Ca(2+)-mediated activation of actomyosin ATPase activity |
|  |  |  | CRK | 0.4733 | involved in several signaling pathways |
|  |  |  | CYTH2 | 0.4463 | promotes the activation of ARF factors through replacement of GDP with GTP |
|  |  |  | PDLIM7 | 0.4855 | act as signal mediators involved in a variety of cellular processes |
|  |  |  | F3 | 0.2947 | as the high-affinity receptor for the coagulation factor VII |
|  |  |  | SWAP70 | 0.4972 | involved in B-cell activation, migration |
|  |  |  | NIACR2 | 0.2313 | G protein-coupled receptors |
|  |  |  | SKIL | 0.3599 | a TGFβ signaling mediator |
|  |  |  | STX11 | 0.4325 | a SNARE protein enriched in cells of the immune system |
|  |  |  | GFPT2 | 0.3788 | involved in regulating the availability of precursors for N- and O-linked glycosylation of proteins |
|  |  |  | NNMT | 0.4267 | crucial role in cellular invasion |
|  |  |  | DSE | 0.4487 | a tumor-rejection antigen |
|  |  |  | PDE4A | 0.4932 | hydrolyzes the second messenger cAMP |
|  |  |  |  |  |  |
|  | **Cytoskeleton** |  | SPG20 | 0.462 | regulating endosomal trafficking and mitochondria function |
|  |  |  | CYLC2 | 0.3175 | play a role in the morphogenesis of the sperm head |
|  |  |  | TCAP | 0.4095 | a substrate of titin kinase; be critical to sarcomere assembly |
|  |  |  | STRC | 0.2471 | associated with the hair bundle of the sensory hair cells in the inner ear |
|  |  |  | ERMN | 0.4548 | a role in cytoskeletal rearrangements |
|  |  |  | ACTA1 | 0.344 | play a role in cell motility, structure and integrity |
|  |  |  | CDC42EP2 | 0.45 | involved in the organization of the actin cytoskeleton |
|  |  |  | CXCR3 | 0.4795 | involved in leukocyte traffic, most notably integrin activation, cytoskeletal changes and chemotactic migration |
|  |  |  | EMD | 0.4879 | a serine-rich nuclear membrane protein; mediates membrane anchorage to the cytoskeleton |
|  |  |  | ODF4 | 0.3699 | involved in sperm tail structure,sperm movement and general organization of cellular cytoskeleton |
|  |  |  | ARHGEF5 | 0.4238 | involved in the control of cytoskeletal organization |
|  |  |  | RIMS2 | 0.3589 | involved in exocytosis; act as scaffold protein |
|  |  |  | MAGI1 | 0.3618 | play a role as scaffolding protein at cell-cell junctions |
|  |  |  | DMD | 0.2042 | anchors the extracellular matrix to the cytoskeleton via F-actin |
|  |  |  | OBSCN | 0.4698 | involved in myofibrillogenesis |
|  |  |  | SPAG4 | 0.3401 | associated with the axoneme in elongating spermatids and epididymal sperm |
|  |  |  |  |  |  |
|  | **Enzymes** |  | PLAU | 0.4845 | a serine protease |
|  |  |  | CYBA | 0.4565 | critical component of the membrane-bound oxidase |
|  |  |  | ACP5 | 0.4413 | involved in osteopontin/bone sialoprotein dephosphorylation |
|  |  |  | SDS | 0.1882 | involved in metabolizing serine and glycine |
|  |  |  | AADACL1 | 0.4924 | involved in organ detoxification by hydrolyzing exogenous organophosphorus compounds |
|  |  |  | OTUD7B | 0.4871 | Hydrolyzes both linear and branched forms of polyubiquitin |
|  |  |  | GAL3ST3 | 0.4285 | transfers a sulfate to position 3 of non-reducing beta-galactosyl residues in N-glycans and core2-branched O-glycans |
|  |  |  | GNMT | 0.4558 | catalyzes the methylation of glycine to form N-methylglycine (sarcosine) with the concomitant production of S-adenosylhomocysteine (AdoHcy) |
|  |  |  | ATP2B3 | 0.4399 | catalyzes the hydrolysis of ATP coupled with the transport of calcium out of the cell |
|  |  |  | CA1 | 0.3675 | hydrates cyanamide to urea |
|  |  |  | PROC | 0.42 | a vitamin K-dependent serine protease |
|  |  |  | GPX3 | 0.4404 | Protects cells and enzymes from oxidative damage |
|  |  |  | PTGES | 0.2884 | a glutathione-dependent prostaglandin E synthase |
|  |  |  | DHRS3 | 0.3952 | catalyze the oxidation/reduction of a wide range of substrates, including retinoids and steroids |
|  |  |  | PGM2L1 | 0.3645 | catalyze the 1,3-bisphosphoglycerate-dependent synthesis of glucose 1,6-bisphosphate and other aldose-bisphosphates |
|  |  |  |  |  |  |
|  | **Biosynthesis of macromolecules** |  | COL9A3 | 0.374 | the major collagen component of hyaline cartilage |
|  |  |  | WFDC8 | 0.2542 | as a protease inhibitor |
|  |  |  | COL4A5 | 0.4751 | the major structural component of glomerular basement membranes (GBM) |
|  |  |  | RBP5 | 0.3849 | intracellular mediator of retinol metabolism |
|  |  |  | A4GALT | 0.3275 | Necessary for the biosynthesis of the Pk antigen of blood histogroup P and the receptor for bacterial verotoxins |
|  |  |  | TAGLN | 0.4503 | a transformation and shape-change sensitive actin cross-linking/gelling protein |
|  |  |  | DERL3 | 0.4999 | involved in the degradation of misfolded glycoproteins in the endoplasmic reticulum |
|  |  |  | HBE1 | 0.4727 | a beta-type chain of early mammalian embryonic hemoglobin |
|  |  |  | KYNU | 0.488 | involved in the biosynthesis of NAD cofactors from tryptophan through the kynurenine pathway |
|  |  |  | GPR84 | 0.305 | important roles in processes from fatty acid metabolism to regulation of the immune system |
|  |  |  | LPIN3 | 0.4286 | regulates fatty acid metabolism |
|  |  |  | LSDP5 | 0.113 | binds to lipid droplets and protects them from lipolytic degradation |
|  |  |  | TCF7L2 | 0.4174 | implicated in blood glucose homeostasis |
